# AHaH Computing–From Metastable Switches to Attractors to Machine Learning

**DOI:** 10.1371/journal.pone.0085175

**Published:** 2014-02-10

**Authors:** Michael Alexander Nugent, Timothy Wesley Molter

**Affiliations:** 1 M. Alexander Nugent Consulting, Santa Fe, New Mexico, United States of America; 2 KnowmTech LLC, Albuquerque, New Mexico, United States of America; 3 Xeiam LLC, Santa Fe, New Mexico, United States of America; University of Adelaide, Australia

## Abstract

Modern computing architecture based on the separation of memory and processing leads to a well known problem called the von Neumann bottleneck, a restrictive limit on the data bandwidth between CPU and RAM. This paper introduces a new approach to computing we call AHaH computing where memory and processing are combined. The idea is based on the attractor dynamics of volatile dissipative electronics inspired by biological systems, presenting an attractive alternative architecture that is able to adapt, self-repair, and learn from interactions with the environment. We envision that both von Neumann and AHaH computing architectures will operate together on the same machine, but that the AHaH computing processor may reduce the power consumption and processing time for certain adaptive learning tasks by orders of magnitude. The paper begins by drawing a connection between the properties of volatility, thermodynamics, and Anti-Hebbian and Hebbian (AHaH) plasticity. We show how AHaH synaptic plasticity leads to attractor states that extract the independent components of applied data streams and how they form a computationally complete set of logic functions. After introducing a general memristive device model based on collections of metastable switches, we show how adaptive synaptic weights can be formed from differential pairs of incremental memristors. We also disclose how arrays of synaptic weights can be used to build a neural node circuit operating AHaH plasticity. By configuring the attractor states of the AHaH node in different ways, high level machine learning functions are demonstrated. This includes unsupervised clustering, supervised and unsupervised classification, complex signal prediction, unsupervised robotic actuation and combinatorial optimization of procedures–all key capabilities of biological nervous systems and modern machine learning algorithms with real world application.

## Introduction

How does nature compute? Attempting to answer this question naturally leads one to consider biological nervous systems, although examples of computation abound in other manifestations of life. Some examples include plants [1]–[5], bacteria [6], protozoan [7], and swarms [8], to name a few. Most attempts to understand biological nervous systems fall along a spectrum. One end of the spectrum attempts to mimic the observed physical properties of nervous systems. These models necessarily contain parameters that must be tuned to match the biophysical and architectural properties of the natural model. Examples of this approach include Boahen’s neuromorphic circuit at Stanford University and their Neurogrid processor [9], the mathematical spiking neuron model of Izhikevich [10] as well as the large scale modeling of Eliasmith [11]. The other end of the spectrum abandons biological mimicry in an attempt to algorithmically solve the problems associated with brains such as perception, planning and control. This is generally referred to as machine learning. Algorithmic examples include support vector maximization [12], *k*-means clustering [13] and random forests [14]. Many approaches fall somewhere along the spectrum between mimicry and machine learning, such as the CAVIAR [15] and Cognimem [16] neuromorphic processors as well as IBM’s *neurosynaptic core* [17]. In this paper we consider an alternative approach outside of the typical spectrum by asking ourselves a simple but important question: How can a brain compute given that it is built of volatile components?

A brain, like all living systems, is a far-from-equilibrium energy dissipating structure that constantly builds and repairs itself. We can shift the standard question from “how do brains compute?” or “what is the algorithm of the brain?” to a more fundamental question of “how do brains build and repair themselves as dissipative attractor-based structures?” Just as a ball will roll into a depression, an attractor-based system will fall into its attractor states. Perturbations (damage) will be fixed as the system reconverges to its attractor state. As an example, if we cut ourselves *we heal*. To bestow this property on our computing technology we must find a way to represent our computing structures as attractors. In this paper we detail how the attractor points of a plasticity rule we call Anti-Hebbian and Hebbian (AHaH) plasticity are computationally complete logic functions as well as building blocks for machine learning functions. We further show that AHaH plasticity can be attained from simple memristive circuitry attempting to maximize circuit power dissipation in accordance with ideas in nonequilibrium thermodynamics.

Our goal is to lay a foundation for a new type of practical computing based on the configuration and repair of volatile switching elements. We traverse the large gap from volatile memristive devices to demonstrations of computational universality and machine learning. The reader should keep in mind that the subject matter in this paper is necessarily diverse, but is essentially an elaboration of these three points:

1. AHaH plasticity emerges from the interaction of volatile competing energy dissipating pathways.
2. AHaH plasticity leads to attractor states that can be used for universal computation and advanced machine learning
3. Neural nodes operating AHaH plasticity can be constructed from simple memristive circuits.

### The Adaptive Power Problem

Through constant dissipation of free energy, living systems continuously repair their seemingly fragile state. A byproduct of this condition is that living systems are intrinsically adaptive at all scales, from cells to ecosystems. This presents a difficult challenge when we attempt to simulate such large scale adaptive networks with modern von Neumann computing architectures. Each adaptation event must necessarily reduce to memory–processor communication as the state variables are modified. The energy consumed in shuttling information back and forth grows in line with the number of state variables that must be continuously modified. For large scale adaptive systems like the brain, the inefficiencies become so large as to make simulations impractical.

As an example, consider that IBM’s recent cat-scale cortical simulation of 1 billion neurons and 10 trillion synapses [18] required 147,456 CPUs, 144 TB of memory, running at 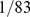 real-time. At a power consumption of 20 W per CPU, this is 2.9 MW. Under perfect scaling, a real-time simulation of a human-scale cortex would dissipate over 7 GW of power. The number of adaptive variables under constant modification in the IBM simulation is orders of magnitude less than the biological counterpart and yet its power dissipation is orders of magnitude larger. Another example from Google to train neural networks on YouTube data roughly doubled the accuracy from previous attempts [19]. The effort took an array of 16,000 CPU cores working at full capacity for 3 days. The model contained 1 billion connections, which although impressive pales in comparison to biology. The average human neocortex contains 150,000 billion connections [20] and the number of synapses in the neocortex is a fraction of the total number of connections in the brain. At 20 W per core, Google’s simulation consumed about 320 kW. Under perfect scaling, a human-scale simulation would dissipate 48 GW of power.

At the core of the adaptive power problem is the energy wasted during memory–processor communication. The ultimate solution to the problem entails finding ways to let memory configure itself, and AHaH computing is one such method.

### The Adaptive Power Solution

Consider two switches, one non-volatile and the other volatile. Furthermore, consider what it takes to change the state of each of these switches, which is the most fundamental act of adaptation or reconfiguration. Abstractly, a switch can be represented as a potential energy well with two or more minima.

In the non-volatile case, sufficient energy must be applied to overcome the barrier potential. Energy must be dissipated in proportion to the barrier height once a switching event takes place. Rather than just the switch, it is also the electrode leading to the switch that must be raised to the switch barrier energy. As the number of adaptive variables increases, the power required to sustain the switching events scales as the total distance needed to communicate the switching events and the square of the voltage.

A volatile switch on the other hand cannot be read without damaging its state. Each read operation lowers the switch barriers and increases the probability of random state transitions. Accumulated damage to the state must be actively repaired. In the absence of repair, the act of reading the state is alone sufficient to induce state transitions. The distance that must be traversed between memory and processing of an adaptation event goes to zero as the system becomes intrinsically adaptive. The act of accessing the memory *becomes* the act of configuring the memory.

In the non-volatile case some process external to the switch (i.e. an algorithm on a CPU) must provide the energy needed to effect the state transition. In the volatile case an external process must *stop* providing the energy needed for state repair. These two antisymmetric conditions can be summarized as: “Stability for free, adaptation for a price” and “adaptation for free, stability for a price”, respectively.

Not only does it make physical sense to build large scale adaptive systems from volatile components but furthermore there is no supporting evidence to suggest it is possible to do the contrary. A brain is a volatile dissipative out-of-equilibrium structure. It is therefore reasonable that a volatile solution to machine learning at low power and high densities exists. The goal of AHaH computing is to find and exploit this solution.

### Historical Background

In 1936, Turing, best known for his pioneering work in computation and his seminal paper ‘On computable numbers’ [21], provided a formal proof that a machine could be constructed to be capable of performing any conceivable mathematical computation if it were representable as an algorithm. This work rapidly evolved to become the computing industry of today. Few people are aware that, in addition to the work leading to the digital computer, Turing anticipated connectionism and neuron-like computing. In his paper ‘Intelligent machinery’ [22], which he wrote in 1948 but was not published until well after his death in 1968, Turing described a machine that consists of artificial neurons connected in any pattern with modifier devices. Modifier devices could be configured to pass or destroy a signal, and the neurons were composed of NAND gates that Turing chose because any other logic function can be created from them.

In 1944, physicist Schrödinger published the book *What is Life?* based on a series of public lectures delivered at Trinity College in Dublin. Schrödinger asked the question: “How can the events in space and time which take place within the spatial boundary of a living organism be accounted for by physics and chemistry?” He described an aperiodic crystal that predicted the nature of DNA, yet to be discovered, as well as the concept of *negentropy* being the entropy of a living system that it exports to keep its own entropy low [23].

In 1949, only one year after Turing wrote ‘Intelligent machinery’, synaptic plasticity was proposed as a mechanism for learning and memory by Hebb [24]. Ten years later in 1958 Rosenblatt defined the theoretical basis of connectionism and simulated the *perceptron*, leading to some initial excitement in the field [25].

In 1953, Barlow discovered neurons in the frog brain fired in response to specific visual stimuli [26]. This was a precursor to the experiments of Hubel and Wiesel who showed in 1959 the existence of neurons in the primary visual cortex of the cat that selectively responds to edges at specific orientations [27]. This led to the theory of receptive fields where cells at one level of organization are formed from inputs from cells in a lower level of organization.

In 1960, Widrow and Hoff developed ADALINE, a physical device that used electrochemical plating of carbon rods to emulate the synaptic elements that they called *memistors* [28]. Unlike memristors, memistors are three terminal devices, and their conductance between two of the terminals is controlled by the time integral of the current in the third. This work represents the first integration of memristive-like elements with electronic feedback to emulate a learning system.

In 1969, the initial excitement with perceptrons was tampered by the work of Minsky and Papert, who analyzed some of the properties of perceptrons and illustrated how they could not compute the XOR function using only local neurons [29]. The reaction to Minsky and Papert diverted attention away from connection networks until the emergence of a number of new ideas, including Hopfield networks (1982) [30], back propagation of error (1986) [31], adaptive resonance theory (1987) [32], and many other permutations. The wave of excitement in neural networks began to fade as the key problem of generalization versus memorization became better appreciated and the computing revolution took off.

In 1971, Chua postulated on the basis of symmetry arguments the existence of a missing fourth two terminal circuit element called a memristor (*memory resistor*), where the resistance of the memristor depends on the integral of the input applied to the terminals [33], [34].

VLSI pioneer Mead published with Conway the landmark text *Introduction to VLSI Systems* in 1980 [35]. Mead teamed with John Hopfield and Feynman to study how animal brains compute. This work helped to catalyze the fields of Neural Networks (Hopfield), Neuromorphic Engineering (Mead) and Physics of Computation (Feynman). Mead created the world’s first neural-inspired chips including an artificial retina and cochlea, which was documented in his book *Analog VLSI Implementation of Neural Systems* published in 1989 [36].

Beinenstock, Cooper and Munro published a theory of synaptic modification in 1982 [37]. Now known as the BCM plasticity rule, this theory attempts to account for experiments measuring the selectivity of neurons in primary sensory cortex and its dependency on neuronal input. When presented with data from natural images, the BCM rule converges to selective oriented receptive fields. This provides compelling evidence that the same mechanisms are at work in cortex, as validated by the experiments of Hubel and Wiesel. In 1989 Barlow reasoned that such selective response should emerge from an unsupervised learning algorithm that attempts to find a factorial code of independent features [38]. Bell and Sejnowski extended this work in 1997 to show that the independent components of natural scenes are edge filters [39]. This provided a concrete mathematical statement on neural plasticity: Neurons modify their synaptic weight to extract independent components. Building a mathematical foundation of neural plasticity, Oja and collaborators derived a number of plasticity rules by specifying statistical properties of the neuron’s output distribution as objective functions. This lead to the principle of *independent component analysis* (ICA) [40], [41].

At roughly the same time, the theory of support vector maximization emerged from earlier work on statistical learning theory from Vapnik and Chervonenkis and has become a generally accepted solution to the generalization versus memorization problem in classifiers [12], [42].

In 2004, Nugent et al. showed how the AHAH plasticity rule is derived via the minimization of a kurtosis objective function and used as the basis of self-organized fault tolerance in support vector machine network classifiers. Thus, the connection that margin maximization coincides with independent component analysis and neural plasticity was demonstrated [43], [44]. In 2006, Nugent first detailed how to implement the AHaH plasticity rule in memristive circuitry and demonstrated that the AHaH attractor states can be used to configure a universal reconfigurable logic gate [45]–[47].

In 2008, HP Laboratories announced the production of Chua’s postulated electronic device, the memristor [48] and explored their use as synapses in neuromorphic circuits [49]. Several memristive devices were previously reported by this time, predating HP Laboratories [50]–[54], but they were not described as memristors. In the same year, Hylton and Nugent launched the Systems of Neuromorphic Adaptive Plastic Scalable Electronics (SyNAPSE) program with the goal of demonstrating large scale adaptive learning in integrated memristive electronics at biological scale and power. Since 2008 there has been an explosion of worldwide interest in memristive devices [55]–[59] device models [60]–[65], their connection to biological synapses [66]–[72], and use in alternative computing architectures [73]–[84].

## Theory

### On the Origins of Algorithms and the 4th Law of Thermodynamics

Turing spent the last two years of his life working on mathematical biology and published a paper titled ‘The chemical basis of morphogenesis’ in 1952 [85]. Turing was likely struggling with the concept that algorithms represent structure, brains and life in general are clearly capable of creating such structure, and brains are ultimately a biological chemical process that emerge from chemical homogeneity. How does complex spatial-temporal structure such as an algorithm emerge from the interaction of a homogeneous collection of units?

Answering this question in a physical sense leads one straight into the controversial 4th law of thermodynamics. The 4th law is is attempting to answer a simple question with profound consequences if a solution is found: If the 2nd law says everything tends towards disorder, why does essentially everything we see in the Universe contradict this? At almost every scale of the Universe we see self-organized structures, from black holes to stars, planets and suns to our own earth, the life that abounds on it and in particular the brain. Non-biological systems such as Benard convection cells [86], tornadoes, lightning and rivers, to name just a few, show us that matter does not tend toward disorder in practice but rather does quite the opposite. In another example, metallic spheres in a non-conducting liquid medium exposed to an electric field will self-organize into fractal dendritic trees [87].

One line of argument is that ordered structures create entropy faster than disordered structures do and self-organizing dissipative systems are the result of *out of equilibrium thermodynamics*. In other words, there may not actually be a distinct 4th law, and all observed order may actually result from dynamics yet to be unraveled mathematically from the 2nd law. Unfortunately this argument does not leave us with an understanding sufficient to allow us to exploit the phenomena in our technology. In this light, our work with AHaH attractor states may provide a clue as to the nature of the 4th law in so much as it lets us construct useful self-organizing and adaptive computing systems.

One particularly clear and falsifiable formulation of the 4th law comes from Swenson in 1989:

“A system will select the path or assembly of paths out of available paths that minimizes the potential or maximizes the entropy at the fastest rate given the constraints [88].”

Others have converged on similar thoughts. For example, Bejan postulated in 1996 that:

“For a finite-size system to persist in time (to live), it must evolve in such a way that it provides easier access to the imposed currents that flow through it [89].”

Bejan’s formulation seems intuitively correct when one looks at nature, although it has faced criticism that it is too vague since it does not say what particle is flowing. We observe that in many cases the particle is either directly a carrier of free energy dissipation or else it gates access, like a key to a lock, to free energy dissipation of the units in the collective. These particles are not hard to spot. Examples include water in plants, ATP in cells, blood in bodies, neurotrophins in brains, and money in economies.

More recently, Jorgensen and Svirezhev have put forward the *maximum power principle* [90] and Schneider and Sagan have elaborated on the simple idea that “nature abhors a gradient” [91]. Others have put forward similar notions much earlier. Morowitz claimed in 1968 that the flow of energy from a source to a sink will cause at least one cycle in the system [91] and Lotka postulated the *principle of maximum energy flux* in 1922 [92].

### The Container Adapts

Hatsopoulos and Keenan’s *law of stable equilibrium* [93] states that:

“When an isolated system performs a process, after the removal of a series of internal constraints, it will always reach a unique state of equilibrium; this state of equilibrium is independent of the order in which the constraints are removed.”

The idea is that a system erases any knowledge about how it arrived in equilibrium. Schneider and Sagan state this observation in their book *Into the Cool: Energy Flow, Thermodynamics, and Life* [91] by claiming: “These principles of erasure of the path, or past, as work is produced on the way to equilibrium hold for a broad class of thermodynamic systems.” This principle has been illustrated by connected rooms, where doors between the rooms are opened according to a particular sequence, and only one room is pressurized at the start. The end state is the same regardless of the path taken to get there. The problem with this analysis is that it relies on an external agent: the door opener.

**Figure 1:**
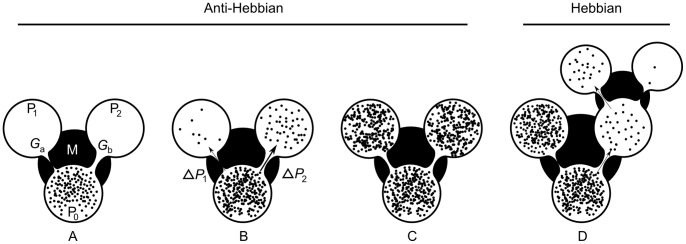
AHaH process. A) A first replenished pressurized container 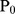 is allowed to diffuse into two non-pressurized empty containers 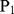 and 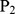 though a region of matter M. B) The gradient 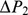 reduces faster than the gradient 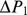 due to the conductance differential. C) This causes 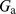 to grow more than 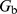, reducing the conductance differential and leading to anti-Hebbian learning. D) The first detectable signal (work) is available at 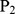 owing to the differential that favors it. As a response to this signal, events may transpire in the environment that open up new pathways to particle dissipation. The initial conductance differential is reinforced leading to Hebbian learning.

We may reformulate this idea in the light of an adaptive container, as shown in Figure 1. A first *replenished* pressurized container 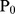 is allowed to diffuse into two non-pressurized empty containers 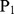 and 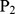 though a region of matter 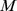. Let us presume that the initial fluid conductance 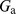 between 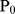 and 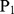 is less than 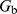. Competition for limited resources within the matter (conservation of matter) enforces the condition that the sum of conductances is constant:
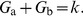
(1)


Now we ask how the container adapts as the system attempts to come to equilibrium. If it is the *gradient* that is driving the change in the conductance, then it becomes immediately clear that the container will adapt in such a way as to erase any initial differential conductance:
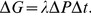
(2)


The gradient 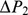 will reduce faster than the gradient 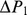 and 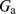 will grow more than 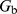. When the system comes to equilibrium we will find that the conductance differential, 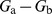 has been reduced.

The sudden pressurization of 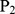 may have an effect on the environment. In the moments right after the flow sets up, the first detectable signal (work) will be available at 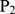 owing to the differential that favors it. As a response to this signal, any number of events could transpire in the environment that open up new pathways to particle dissipation. The initial conductance differential will be reinforced as the system rushes to equalize the gradient in this newly discovered space. Due to conservation of adaptive resources (Equation 1), an increase in 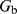 will require a drop in 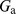, and vice versa. The result is that as 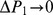, 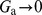, 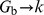 and the system selects one pathway over another. The process illustrated in Figure 1 creates structure so long as new sinks are constantly found and a constant particle source is available.

We now map this thermodynamic process to anti-Hebbian and Hebbian (AHaH) plasticity and show that the resulting attractor states support universal algorithms and broad machine learning functions. We furthermore show how AHaH plasticity can be implemented via physically adaptive memristive circuitry.

### Anti-Hebbian and Hebbian (AHaH) Plasticity

The thermodynamic process outlined above can be understood more broadly as: (1) particles spread out along all available pathways through the environment and in doing so erode any differentials that favor one branch over the other, and (2) pathways that lead to dissipation (the flow of the particles) are stabilized. Let us first identify a synaptic weight, 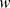, as the differential conductance formed from two energy dissipating pathways:
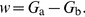
(3)


We can now see that the synaptic weight possess state information. If 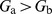 the synapse is positive and if 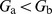 then it is negative. With this in mind we can explicitly define AHaH learning:

- Anti-Hebbian (erase the path): Any modification to the synaptic weight that reduces the probability that the synaptic state will remain the same upon subsequent measurement.
- Hebbian (select the path): Any modification to the synaptic weight that increases the probability that the synaptic state will remain the same upon subsequent measurement.

Our use of Hebbian learning follows a standard mathematical generalization of Hebb’s famous postulate:

“When an axon of cell A is near enough to excite B and repeatedly or persistently takes part in firing it, some growth process or metabolic change takes place in one or both cells such that A’s efficiency, as one of the cells firing B, is increased [24].”

Hebbian learning can be represented mathematically as 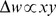, where 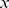 and 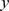 are the activities of the pre- and post-synaptic neurons and 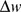 is the change to the synaptic weight between them. Anti-Hebbian learning is the negative of Hebbian: 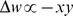. Notice that intrinsic to this mathematical definition is the notion of state. The pre- and post-synaptic activities as well as the weight may be positive or negative. We achieve the notion of state in our physical circuits via differential conductances (Equation 3).

### Linear Neuron Model

To begin our mapping of AHaH plasticity to computing and machine learning systems we use a standard linear neuron model. The choice of a linear neuron is motivated by the fact that they are ubiquitous in machine learning and also because it is easy to achieve the linear sum function in a physical circuit, since currents naturally sum.

The inputs 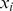 in a linear model are the outputs from other neurons or spike encoders (to be discussed). The weights 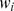 are the strength of the inputs. The larger 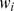, the more 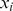 affects the neuron’s output. Each input 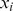 is multiplied by a corresponding weight 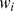 and these values, combined with the bias 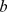, are summed together to form the output 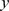:
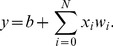
(4)


The weights and bias change according to AHaH plasticity, which we further detail in the sections that follow. The AHaH rule acts to *maximize the margin* between positive and negative classes. In what follows, *AHaH nodes* refer to linear neurons implementing the AHaH plasticity rule.

### AHaH Attractors Extract Independent Components

What we desire is a mechanism to extract the underlying building blocks or *independent components* of a data stream, irrespective of the number of discrete channels those components are communicated over. One method to accomplish this task is independent component analysis. The two broadest mathematical definitions of independence as used in ICA are (1) minimization of mutual information between competing nodes and (2) maximization of non-Gaussianity of the output of a single node. The non-Gaussian family of ICA algorithms uses negentropy and kurtosis as mathematical objective functions from which to derive a plasticity rule. To find a plasticity rule capable of ICA we can minimize a kurtosis objective function over the node output activation. The result is ideally the opposite of a peak: a bimodal distribution. That is, we seek a hyperplane that separates the input data into two classes resulting in two distinct *positive* and *negative* distributions. Using a kurtosis objective function, it can be shown that a plasticity rule of the following form emerges [43]:
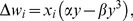
(5)where 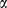 and 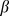 are constants that control the relative contribution of Hebbian and anti-Hebbian plasticity, respectively. Equation 5 is one form of many that we call the *AHaH rule*. The important functional characteristics that Equation 5 shares with all the other forms is that as the magnitude of the post-synaptic activation grows, the weight update transitions from Hebbian to anti-Hebbian learning.

### AHaH Attractors Make Optimal Decisions

An AHaH node is a hyperplane attempting to bisect its input space so as to make a binary decision. There are many hyperplanes to choose from and the question naturally arises as to which one is best. The generally agreed answer to this question is “the one that maximizes the separation (margin) of the two classes.” The idea of *maximizing the margin* is central to support vector machines, arguably one of the more successful machine learning algorithms. As demonstrated in [43], [44], as well as the results of this paper, the attractor states of the AHaH rule coincide with the maximum-margin solution.

### AHaH Attractors Support Universal Algorithms

Given a discrete set of inputs and a discrete set of outputs it is possible to account for all possible transfer functions via a logic function. Logic is usually taught as small two-input gates such as NAND and OR. However, when one looks at a more complicated algorithm such as a machine learning classifier, it is not so clear that it is performing a logic function. As demonstrated in following sections, AHaH attractor states are computationally complete logic functions. For example, when robotic arm actuation or prediction is demonstrated, self-configuring logic functions is also being demonstrated.

In what follows we will be adopting a *spike encoding*. A spike encoding consists of either a spike (1) or no spike (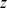). In digital logic, the state ‘0’ is opposite or complimentary to the state ‘1’ and it can be communicated. One cannot communicate a pulse of *nothing* (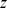). For this reason, we refer to a spike as ‘1’ and no spike as a ‘
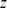
’ or *floating* to avoid this confusion. Furthermore, the output of an AHaH node can be positive or negative and hence possess a *state*. We can identify these positive and negative output states as logical outputs, for example the standard logical ‘1’ is *positive* and ‘0’ is *negative*.

Let us analyze the simplest possible AHaH node; one with only two inputs. The three possible input patterns are:
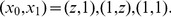
(6)


**Figure 2:**
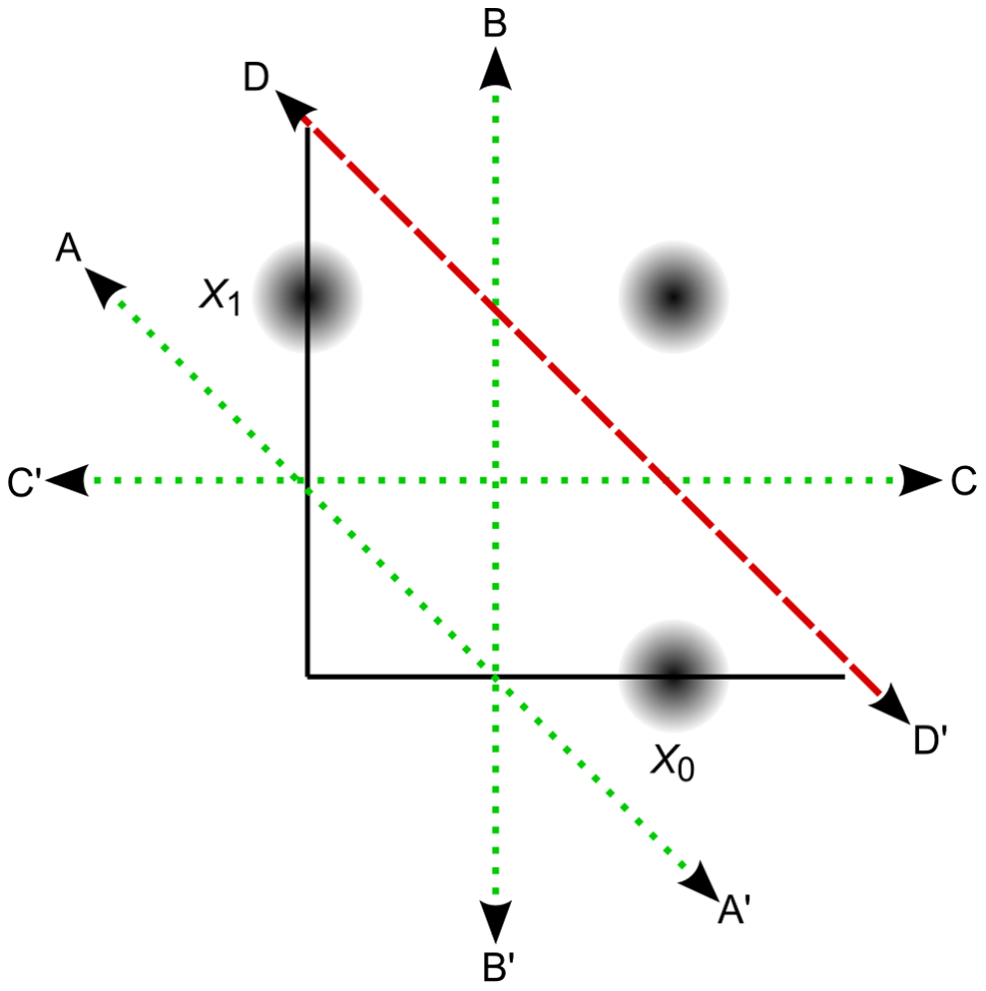
Attractor states of a two-input AHaH node. The AHaH rule naturally forms decision boundaries that maximize the margin between data distributions (black blobs). This is easily visualized in two dimensions, but it is equally valid for any number of inputs. Attractor states are represented by decision boundaries A, B, C (green dotted lines) and D (red dashed line). Each state has a corresponding anti-state: 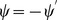. State A is the null state and its occupation is inhibited by the bias. State D has not yet been reliably achieved in circuit simulations.

Stable synaptic states will occur when the sum over all weight updates is zero. We can plot the AHaH node’s stable decision boundary on the same plot with the data that produced it. This can be seen in Figure 2, where decision boundaries A, B and C are labeled. Although the D state is theoretically achievable, it has been difficult to achieve in circuit simulations, and for this reason we exclude it as an available state. Note that every state has a corresponding anti-state. The AHaH plasticity is a local update rule that is attempting to maximize the margin between opposing positive and negative data distributions. As the positive distribution pushes the decision boundary away (making the weights more positive), the magnitude of the positive updates decreases while the magnitude of the opposing negative updates increases. The net result is that strong attractor states exist when the decision boundary can cleanly separate a data distribution.

We refer to the A state as the null state. The null state occurs when an AHaH node assigns the same weight value to each synapse and outputs the same state for every pattern. The null state is mostly useless computationally, and its occupation is inhibited by bias weights. Through strong anti-Hebbian learning, the bias weights force each neuron to split the output space equally. As the neuron *locks on* to a stable bifurcation, the effect of the bias weights is minimized and the decision margin is maximized via AHaH learning on the input weights.

**Figure 3:**
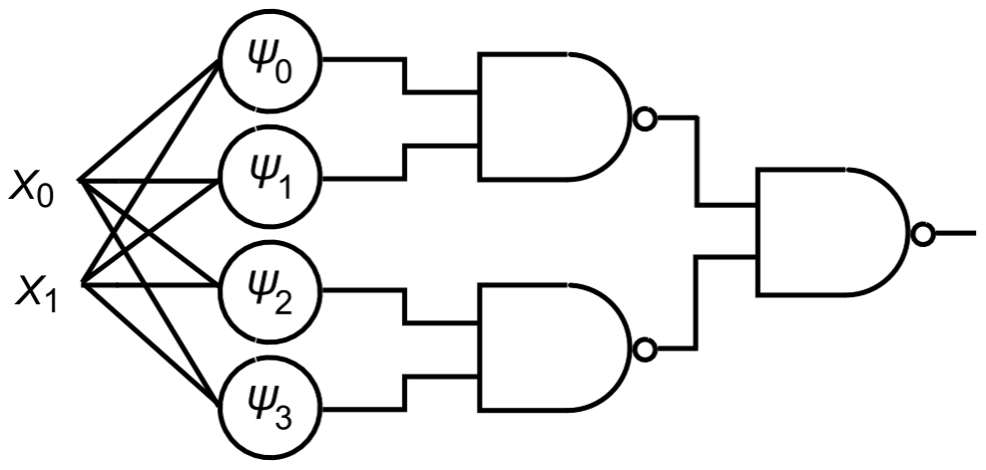
Universal reconfigurable logic. By connecting the output of AHaH nodes (circles) to the input of static NAND gates, one may create a universal reconfigurable logic gate by configuring the AHaH node attractor states (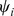). The structure of the data stream on binary encoded channels 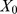 and 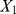 support AHaH attractor states 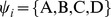 (Figure 2). Through configuration of node attractor states the logic function of the circuit can be configured and all logic functions are possible. If inputs are represented as a spike encoding over four channels then AHaH node attractor states can attain all logic functions without the use of NAND gates.

Recall Turing’s idea of a network of NAND gates connected by *modifier devices* as mentioned in the Historical Background section. The AHaH nodes extract independent component states, the *alphabet* of the data stream. As illustrated in Figure 3, by providing the sign of the output of AHaH nodes to static NAND gates, a universal reconfigurable logic gate is possible. Configuring the AHaH attractor states, 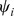, configures the logic function. We can do even better than this however.

We can achieve all logic functions directly (without NAND gates) if we define a *spike logic* code, where 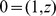 and 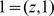, as shown in Table 1. As any algorithm or procedure can be attained from combinations of logic functions, AHaH nodes are building blocks from which any algorithm can be built. This analysis of logic is necessary to prove that AHaH attractor states can support any algorithm, not that AHaH computing is intended to replace modern methods of high speed digital logic.

**Table 1:** Spike logic patterns.

Logic Pattern	Spike Logic Pattern
(0, 0)	(1, *z*, 1, *z*)
(0, 1)	(1, *z*, *z*, 1)
(1, 0)	(*z*, 1, 1, *z*)
(1, 1)	(*z*, 1, *z*, 1)

Digital logic states ‘0’ and ‘1’ across two input lines are converted to a spike encoding across four input lines. A spike encoding consists of either spikes (1) or no spikes (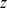). This encoding insures that the number of spikes at any given time is constant.

### AHaH Attractors are Bits

Every AHaH attractor consists of a state/anti-state pair that can be configured and therefore appears to represent a *bit*. In the limit of only one synapse and one input line activation, the state of the AHaH node is the state of the synapse just like a typical bit. As the number of simultaneous inputs grows past one, the *AHaH bit* becomes a collective over all interacting synapses. For every AHaH attractor state that outputs a ‘1’, for example, there exists an equal and opposite AHaH attractor state that will output a ‘−1’. The state/anti-state property of the AHaH attractors follows mathematically from ICA, since ICA is in general not able to uniquely determine the sign of the source signals. The AHaH bits open up the possibility of configuring populations to achieve computational objectives. We take advantage of AHaH bits in the AHaH clustering and AHaH motor controller examples presented later in this paper. It is important to understand that AHaH attractor states are a reflection of the underlying statistics of the data stream and cannot be fully understood as just the collection of synapses that compose it. Rather, it is both the collection of synapses and also the structure of the information that is being processed that result in an AHaH attractor state. If we equate the data being processed as a sequence of measurements of the AHaH bit’s state, we arrive at an interesting observation: the act of measurement not only effects the state of the AHaH bit, it actually *defines* it. Without the data structure imposed by the sequence of measurements, the state would simply not exist. This bears some similarity to ideas that emerge from quantum mechanics.

### AHaH Memristor Circuit

**Figure 4:**
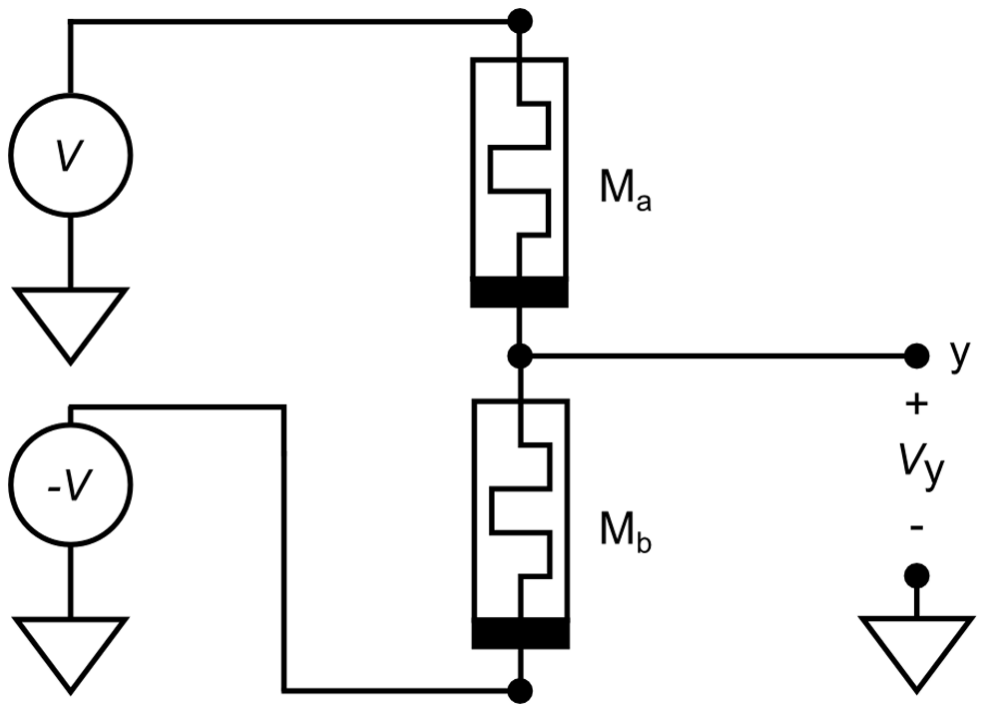
A differential pair of memristors forms a synapse. A differential pair of memristors is used to form a synaptic weight, allowing for both a sign and magnitude. The bar on the memristor is used to indicate polarity and corresponds to the lower potential end when driving the memristor into a higher conductance state. 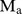 and 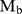 form a voltage divider causing the voltage at node y to be some value between 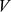 and 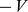. When driven correctly in the absence of Hebbian feedback a synapse will evolve to a symmetric state where 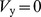 V, alleviating issues arising from device inhomogeneities.

Although we discuss a *functional* or *mathematical* representation of the AHaH node, AHaH computing necessarily has its foundation in a physical embodiment or circuit. The AHaH rule is achievable if one provides for competing adaptive dissipating pathways. The modern memristor provides us with just such an adaptive pathway. Two memristors provide us with two competing pathways. While some neuromorphic computing research has focused on exploiting the synapse-like behavior of a single memristor [68], [83] or using two serially connected memristive devices with different polarities [67], we implement synaptic weights via a differential pair of memristors with the same polarities (Figure 4) [45]–[47] acting as competing dissipation pathways.

The circuits capable of achieving AHaH plasticity can be broadly categorized by the electrode configuration that forms the differential synapses as well as how the input activation (current) is converted to a feedback voltage that drives unsupervised anti-Hebbian learning [46], [47]. Synaptic currents can be converted to a feedback voltage statically (resistors or memristors), dynamically (capacitors), or actively (operational amplifiers). Each configuration requires unique circuitry to drive the electrodes so as to achieve AHaH plasticity, and multiple driving methods exist. The result is that a very large number of AHaH circuits exist, and it is well beyond the scope of this paper to discuss all configurations. Herein, a ‘2-1’ two-phase circuit configuration is introduced because of its compactness and because it is amenable to mathematical analysis.

**Figure 5:**
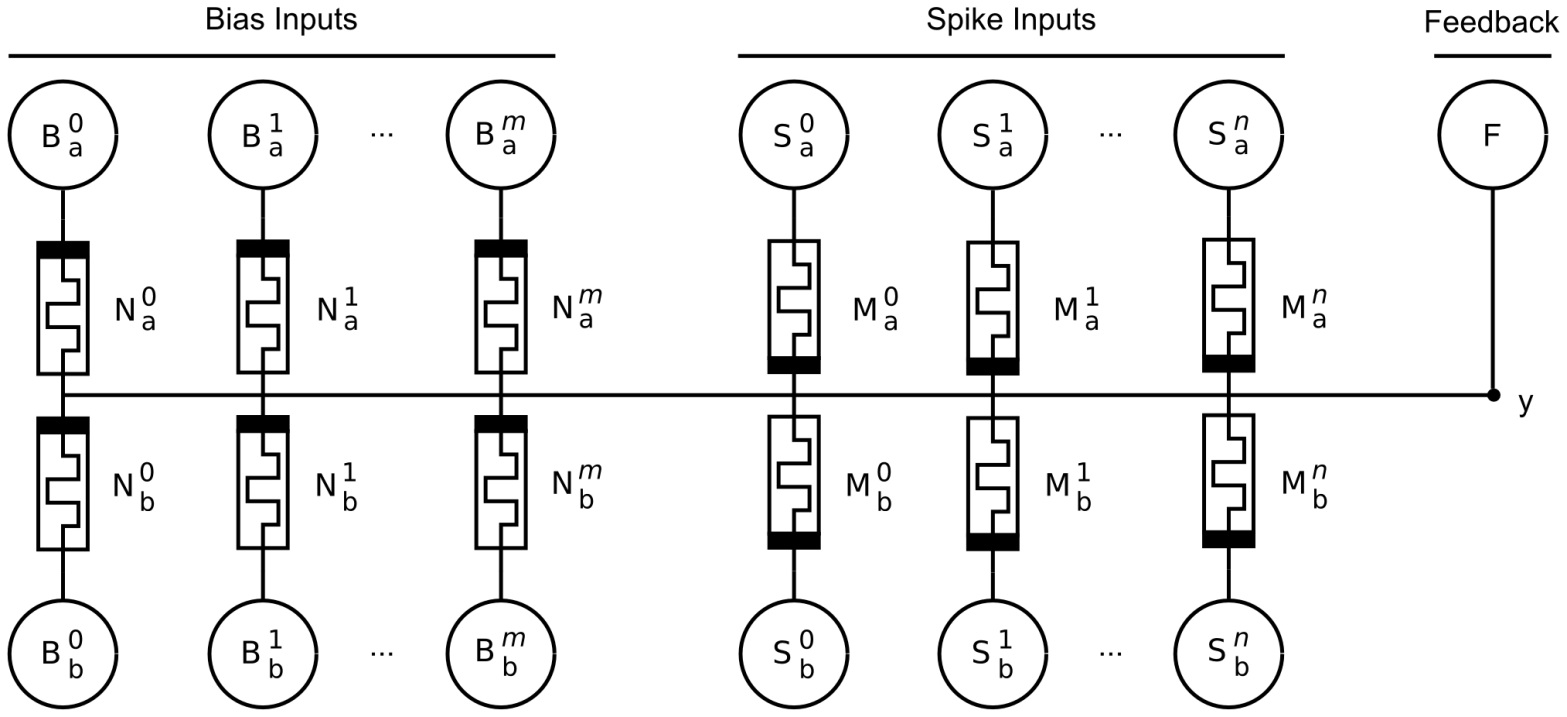
AHaH 2-1 two-phase circuit diagram. The circuit produces an analog voltage signal on the output at node y given a spike pattern on its inputs labeled 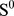, 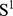 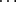, 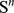. The bias inputs 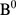, 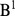 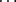, 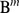 are equivalent to the spike pattern inputs except that they are always active when the spike pattern inputs are active. F is a voltage source used to implement supervised and unsupervised learning via the AHaH rule. The polarity of the memristors for the bias synapse(s) is inverted relative to the input memristors. The output voltage, 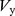, contains both state (positive/negative) and confidence (magnitude) information.

The functional objective of the AHaH circuit shown in Figure 5 is to produce an analog output on electrode y, given an arbitrary spike input of length 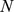 with 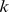 active inputs and 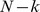 inactive (floating) inputs. The circuit consists of one or more memristor pairs (synapses) sharing a common electrode labeled y. Driving voltage sources are indicated with circles and labeled with an S, B or F, referring to spike, bias, or feedback respectively. The individual driving voltage sources for spike inputs of the AHaH circuit are labeled 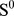, 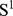 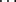, 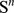. The driving voltage sources for bias inputs are labeled 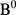, 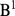 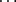, 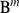. The driving voltage source for supervised and unsupervised learning is labeled F. The subscript values a and b indicate the positive and negative dissipative pathways, respectively.

During the read phase, driving voltage sources 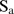 and 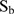 are set to 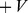 and 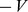 respectively for all 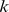 active inputs. Inactive S inputs are left floating. The number of bias inputs to drive, 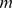, is fixed or a function of 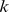 and driving voltage sources 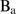 and 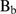 are set to 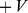 and 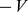 respectively for all bias pairs. The combined conductance of the active inputs and biases produce an output voltage on electrode y. This analog signal contains useful confidence information and can be digitized via the 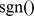 function to either a logical 1 or a 0, if desired.

During the write phase, driving voltage source F is set to either 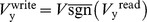 (unsupervised) or 
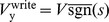
 (supervised), where 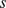 is an externally applied teaching signal. The polarity of the driving voltage sources 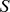 and 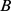 are inverted to 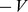 and 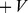. The polarity switch causes all active memristors to be driven to a less conductive state, counteracting the read phase. If this dynamic counteraction did not take place, the memristors would quickly saturate into their maximally conductive states, rendering the synapses useless.

A more intuitive explanation of the above feedback cycle is that “the winning pathway is rewarded by not getting decayed.” Each synapse can be thought of as two competing energy dissipating pathways (positive or negative evaluations) that are building structure (differential conductance). We may apply reinforcing Hebbian feedback by (1) allowing the winning pathway to dissipate more energy or (2) forcing the decay of the losing pathway. If we chose method (1) then we must at some future time ensure that we decay the conductance before device saturation is reached. If we chose method (2) then we achieve both decay and reinforcement at the same time.

### AHaH Rule from Circuit Derivation

Without significant demonstrations of utility there is little motivation to pursue a new form of computing. Our functional model abstraction is necessary to reduce the computational overhead associated with simulating circuits and enable large scale simulations that tackle benchmark problems with real world utility. In this section, we derive the AHaH plasticity rule again, but instead of basing it on statistical independent components as in the derivation of Equation 5, we derive it from simple circuit physics.

During the read phase, simple circuit analysis shows that the voltage on the electrode labeled y in the circuit shown in Figure 5 is:
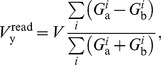
(7)where 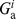 and 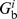 are the conductances of the 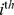 memristors for the positive and negative dissipative pathways, respectively. The driving voltage sources 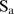 and 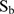 as well as 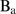 and 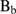 are set to 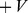 and 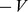 for all 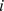 active inputs and bias pairs.

During the write phase the driving voltage source F is set according to either a supervisory signal or in the unsupervised case, the anti-signum of the previous read voltage:
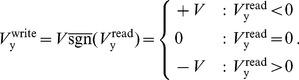
(8)


We may adapt Equation 2 by replacing pressure with voltage:
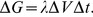
(9)


**Figure 6:**
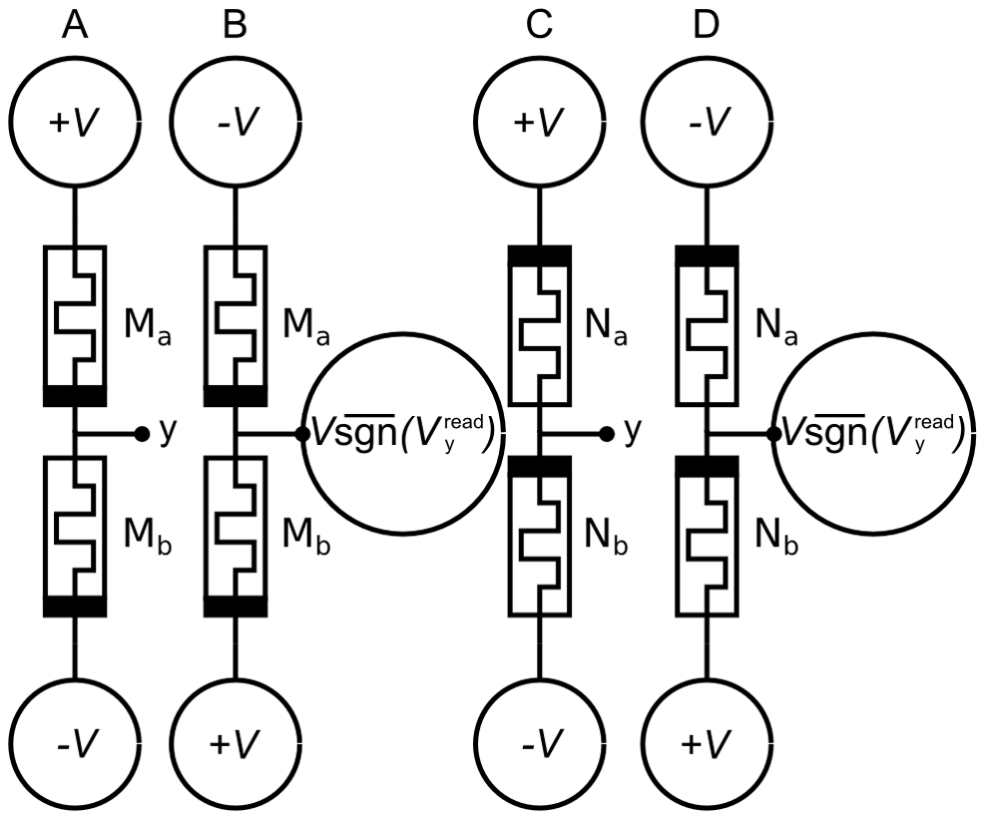
Circuit voltages across memristors during the read and write phases. A) Voltages during read phase across spike input memristors. B) Voltages during write phase across spike input memristors. C) Voltages during read phase across bias memristors. D) Voltages during write phase across bias memristors.

Using Equation 9, the change to memristor conductances over the read and write phases is given in Table 2 and corresponds to the circuits of Figure 6. There are a total of four possibilities because of the two phases and the fact that the polarities of the bias memristors are inverted relative to the spike input memristors. Driving voltage source F is set to 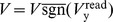 during the write phase for both spike and bias inputs. The terms in Table 2 can be combined to show the total update to the input memristors over the read and write cycle:
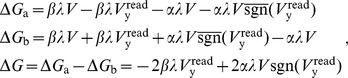
(10)and likewise for the bias memristors:

**Table 2:** Memristor conductance updates during the read and write cycle.

	Input Memristors		Bias Memristors	
	Read	Write	Read	Write
	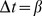	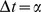	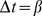	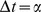
	Accumulate	Decay	Decay	Accumulate
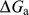	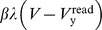	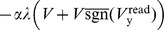	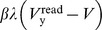	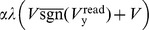
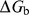	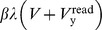	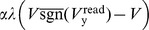	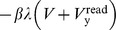	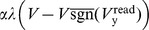

Both input and bias memristors are updated during one read/write cycle. During the read phase the active input memristors increase in conductance (accumulate) while the bias memristors decrease in conductance (decay). During the write phase the active input memristors decrease in conductance while the bias memristors increase in conductance. The changes in memristor conductances, 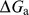 and 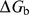, for the memristor pairs are listed for all four cases.



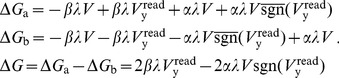
(11)The quantity 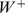, which we call the weight conjugate, remains constant due to competition for limited feedback:
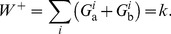
(12)


The output voltage during the read phase reduces to:
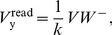
(13)where we have used the substitution:



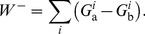
(14)We identify the quantity 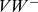 as the standard linear sum over the active weights of the node (Equation 4). Furthermore, we identify the change of the 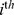 weight as:
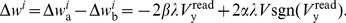
(15)


By absorbing 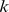, 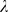 and the two constant 2s into the 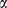 and 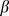 constants we arrive at the functional form *Model A* of the AHaH rule:
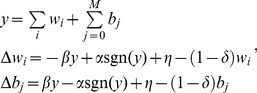
(16)where 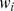 is the 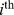 spike input weight, 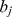 is the 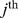 bias weight, and 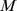 is the total number of biases. To shorten the notation we make the substitution 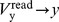. Also note that the quantity 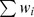 is intended to denote the sum over the active (spiking) inputs. The noise variable 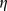 (normal Gaussian) and the decay variable 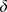 account for the underlying stochastic nature of the memristive devices.

Model A is an approximation that is derived by making simplifying assumptions that include linearization of the update and non-saturation of the memristors. However, when a weight reaches saturation, 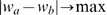, it becomes resistant to Hebbian modification since the weight differential can no longer be increased, only decreased. This has the desirable effect of inhibiting null state occupation. However, it also means that functional Model A is not sufficient to account for these anti-Hebbian forces that grow increasingly stronger as weights near saturation. The result is that Model A leads to strange attractor dynamics and weights that can (but may not) grow without bound, a condition that is clearly unacceptable for a functional model and is not congruent with the circuit.

To account for the growing effect of anti-Hebbian forces we can make a modification to the bias weight update, and we call the resulting form functional *Model B*:
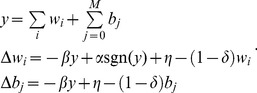
(17)


The purpose of a functional model is to capture equivalent function with minimal computational overhead so that we may pursue large scale application development on existing technology without incurring the computational cost of circuit simulations. We justify the use of Model B because simulations prove it is a close functional match to the circuit, and it is computationally less expensive than Model A. However, it can be expected that better functional forms exist. Henceforth, any reference to the *functional* model refers to Model B.

Finally, in cases where supervision is desired, the sign of the Hebbian feedback may be modulated by an external supervisory signal, 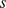, rather than the evaluation state y:
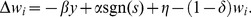
(18)


Compare Equation 17 to Equation 5. Both our functional models as well as the form of Equation 5 converge to functionally similar attractor states. The common characteristic between both forms is a transition from Hebbian to anti-Hebbian learning, as the magnitude of node activation, 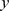, grows large. This transition insures stable AHaH attractor states.

### Generalized Memristive Device Model

Note that AHaH computing is not constrained to just one particular memristive device; any memristive device can be used as long as it meets the following criteria: (1) it is incremental and (2) its state change is voltage dependent. In order to simulate the proposed AHaH node circuit shown in Figure 5, a memristive device model is therefore needed. An effective memristive device model for our use should satisfy several requirements. It should accurately model the device behavior, it should be computationally efficient, and it should model as many different devices as possible. Many memristive device models exist, but we felt compelled to create another one which modeled a wider range of devices and, in particular, shows a transition from stochastic binary to incremental analog properties. Any device that can be manufactured to have electronic behavioral characteristics fitting to our model should be considered a viable component for building AHaH computing devices.

In our proposed semi-empirical model, the total current through the device comes from both a memory-dependent current component, 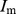, and a Schottky diode current, 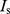 in parallel:
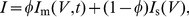
(19)where 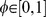. A value of 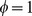 represents a device that contains no Schottky diode effects.

The Schottky component, 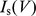, follows from the fact that many memristive devices contain a Schottky barrier formed at a metal–semiconductor junction [48], [63], [68], [94]. The Schottky component is modeled by forward bias and reverse bias components as follows:
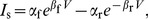
(20)where 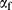, 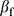, 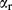, and 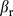 are positive valued parameters setting the exponential behavior of the forward and reverse biases exponential current flow across the Schottky barrier.

The memory component of our model, 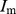, arises from the notion that memristors can be represented as a collection of conducting channels that switch between states of differing resistance. The channels could be formed from molecular switches, atoms, ions, nanoparticles or more complex composite structures. Modification of device resistance is attained through the application of an external voltage gradient that causes the channels to transition between conducting and non-conducting states. As the number of channels increases, the memristor will become more incremental as it acquires the ability to access more states. By modifying the number of channels we may cover a range of devices from binary to incremental. We treat each channel as a *metastable switch* (MSS) and the conductance of a collection of metastable switches capture the memory effect of the memristor.

**Figure 7:**
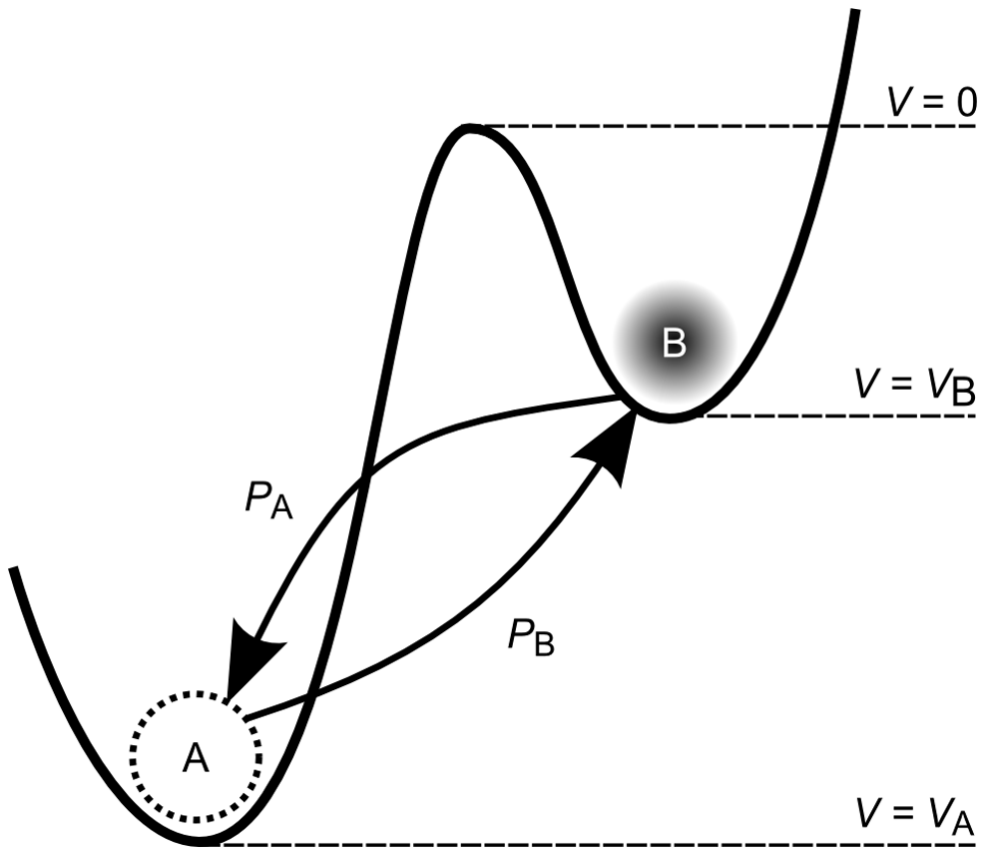
Generalized Metastable Switch (MSS). An MSS is an idealized two-state element that switches probabilistically between its two states as a function of applied voltage bias and temperature. The probability that the MSS will transition from the B state to the A state is given by 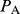, while the probability that the MSS will transition from the A state to the B state is given by 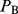. We model a memristor as a collection of 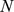 MSSs evolving over discrete time steps.

An MSS possesses two states, A and B, separated by a potential energy barrier as shown in Figure 7. Let the barrier potential be the reference potential 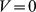. The probability that the MSS will transition from the B state to the A state is given by 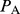, while the probability that the MSS will transition from the A state to the B state is given by 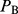. The transition probabilities are modeled as:
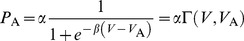
(21)and
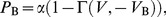
(22)where 
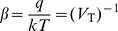
. Here, 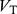 is the thermal voltage and is equal to approximately 26 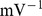 at 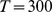 K, 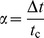 is the ratio of the time step period 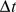 to the characteristic time scale of the device, 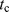, and 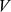 is the voltage across the switch. The probability 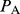 is defined as the positive-going direction, so that a positive applied voltage increases the chances of occupying the A state. An MSS possesses utility in an electrical circuit as an adaptive element so long as these conductances differ. Each state has an intrinsic electrical conductance given by 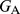 and 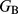. The convention is that 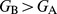. Note that the logistic function 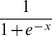 is similar to the hyperbolic-sign function used in other memristive device models including the nonlinear ion-drift, the Simmons tunnel barrier, the threshold adaptive models, and physics-based models [64], [95]–[98]. Our use of the logistic function follows simply from the requirement that probabilities must be bounded between 0 and 1.

We model a memristor as a collection of 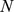 MSSs evolving in discrete time steps, 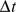. The total memristor conductance is given by the sum over each MSS:
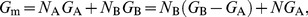
(23)where 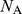 is the number of MSSs in the A state, 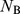 is the number of MSSs in the B state and 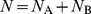.

At each time step some subpopulation of the MSSs in the A state will transition to the B state, while some subpopulation in the B state will transition to the A state. The probability that 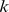 MSSs will transition out of a population of 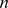 MSSs is given by the binomial distribution:
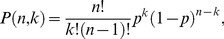
(24)where 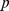 is the probability a MSS will transition states. As 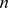 becomes large we may approximate the binomial distribution with a normal distribution:
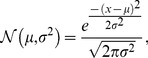
(25)where 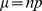 and 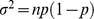.

We model the change in conductance of a memristor as a probabilistic process where the number of switches that transition between A and B states is picked from a normal distribution with a center at 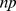 and variance 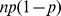, and where the state transition probabilities are given by Equations 21 and 22.

The update to the memristor conductance is given by the contribution from two random variables picked from two normal distributions:
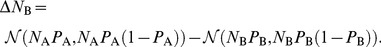
(26)


The final update to the conductance of the memristor is then given by:
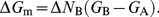
(27)


Reducing the number of MSSs in the model will reduce the averaging effects and cause the memristor to behave in a more stochastic way. As the number of MSSs becomes small, the normal approximation to the binomial distribution breaks down. However, our desired operating regime of many metastable switches, and hence incremental behavior, is within the acceptable bounds of the approximation.

## Methods

All experiments are software based, and they involve the simulation of AHaH nodes in various configurations to perform various adaptive learning tasks. The source code for the experiments is written in the Java programming language and can be obtained from a Git repository linked to from Xeiam LLC’s main web page at http://xeiam.com under the AHaH! project. The code used for the experiments in this paper is tagged as *PLOS_AHAH* on the *master* branch giving a pointer to the exact code used for this paper. The specific programs for each experiment are clearly identified at the end of each experiment description in the methods section. Further details about the programs and the relevant program parameters can be found in the source code itself in the form of comments.

There are two distinct models used for the simulation experiments: functional and circuit. The simulations based on the functional model use functional Model B as described above. The simulations based on the circuit model use ideal electrical circuit components and the generalized model for memristive devices. Nonideal behaviors such as parasitic impedances are not included in the circuit simulation experiments. We want to emphasize that at this stage we are attempting to cross the considerable divide between memristive electronics and general machine learning by defining a theoretical methodology for computing with dissipative attractor states. By focusing on nonideal circuit behavior at this stage we risk obfuscating what is otherwise a theory with minimal complexity.

### Generalized Memristive Device Model

By adjusting the free variables in the generalized memristive device model and comparing the subsequent current-voltage hysteresis loops to four real world memristive device I–V data, matching model parameters were determined as shown in Table 3. The devices include the Ag-chalcogenide [55], AIST [99], GST [70], and WO*_x_* [63] devices, and they represent a wide spectrum of incremental memristive devices found in recent publications exhibiting diverse characteristics. All simulations in this paper involving AHaH node circuitry use the memristor model parameters of the Ag-chalcogenide device, unless otherwise noted. The remaining three are presented in support of our general model.

**Table 3:** General memristive device model parameters fit to various devices.

Device	*t*_c_ [ms]	*G*_A_ [mS]	*G*_B_ [mS]	*V*_A_ [V]	*V*_B_ [V]	*φ*	*α*_f_	*β*_f_	*α*_r_	*β*_r_
Ag-chalc	0.32	8.7	0.91	0.17	0.22	1	–	–	–	–
AIST	0.15	40	10	.23	.25	1	–	–	–	–
GST	0.42	.12	1.2	.9	0.6	0.7	5×10^−3^	3.0	5×10^−3^	3.0
WO*_x_*	0.80	.025	0.004	0.8	1.0	.55	1×10^−9^	8.5	22×10^−9^	6.2

The devices used to test our general memristive device model include the Ag-chalcogenide, AIST, GST, and WO*_x_* devices. The parameters in this table were determined by comparing the model response to a simulated sinusoidal or triangle-wave voltage to real I–V data of physical devices.

**Figure 8:**
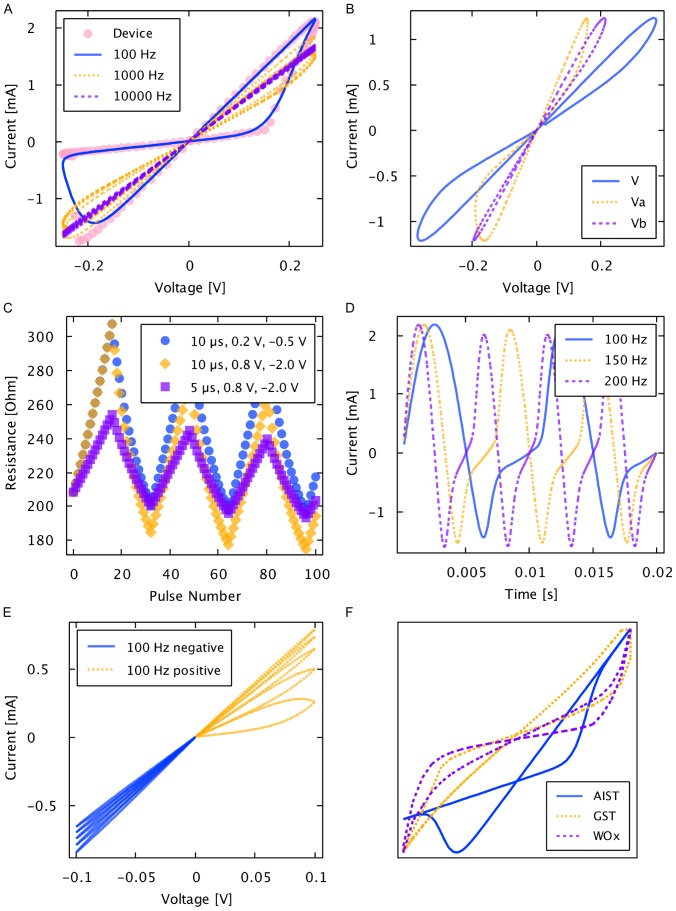
Generalized memristive device model simulations. A) Solid line represents the model simulated at 100 Hz and dots represent the measurements from a physical Ag-chalcogenide device from Boise State University. Physical and predicted device current resulted from driving a sinusoidal voltage of 0.25 V amplitude at 100 Hz across the device. B) Simulation of two series-connected arbitrary devices with differing model parameter values. C) Simulated response to pulse trains of {10 *μ*s, 0.2 V, −0.5 V}, {10 *μ*s, 0.8 V, −2.0 V}, and {5 *μ*s, 0.8 V, −2.0 V} showing the incremental change in resistance in response to small voltage pulses. D) Simulated time response of model from driving a sinusoidal voltage of 0.25 V amplitude at 100 Hz, 150 Hz, and 200 Hz. E) Simulated response to a triangle wave of 0.1 V amplitude at 100 Hz showing the expected incremental behavior of the model. F) Simulated and scaled hysteresis curves for the AIST, GST, and WO*_x_* devices (not to scale).

Figure 8A shows the hysteresis curve of the model and raw Ag-chalcogenide device data driven at 100 Hz with a sinusoidal voltage of 0.25 V amplitude. Additional 1000 Hz and 10 kHz simulations are also shown. The predicted behavior of the model shows a good fit to the physical Ag-chalcogenide device. In fact the model is arguably better than other models (linear ion drift and nonlinear ion drift) tested for a similar device in [61]. Figure 8B shows the predicted response of two series-connected arbitrary memristive devices with differing parameters driven by the sinusoidal voltage as in 8A. The simulation of two devices in series (Figure 4) as shown in Figure 8B also displayed expected characteristics and agrees with results in [100] where the linear ion drift model was used. Experiments have not yet been carried out on physical devices to verify this. Figure 8C shows the incremental pulsed resistance change of a single Ag-chalcogenide modeled device for three different pulse train configurations. The three different pulse trains were chosen to show that by changing both the pulse width or the pulse voltage, the modeled behavior is predicted as expected. Figure 8D shows the time response of the Ag-chalcogenide modeled device at frequencies of 100 Hz, 150 Hz, and 200 Hz. Figure 8E shows the simulated response of the Ag-chalcogenide modeled device to a triangle wave of both +0.1 V and −0.1 V amplitude at 100 Hz designed to show the expected incremental prediction of the model. Figure 8F shows additional model fits to the AIST, GST, and WO*_x_* devices. As demonstrated, our model can be applied to a wide range of memristive devices from Chalcogenides to metal-oxides and more. The source code for these simulations is in *AgChalcogenideHysteresisPlotA.java*, *AgChalcogenideHysteresisPlotB.java*, *AgChalcogenidePulseTrainPlotC*, *AgChalcogenideTimePlotD*, *AgChalcogenideTrianglePlotE*, *AgInSbTeHysteresisPlot.java*, *GSTHysteresisPlot.java*, and *PdWO3WHysteresisPlot.java*.

When it comes time to manufacture AHaH node circuitry, an ideal memristor will be chosen taking into consideration many properties. It is likely that some types of memristors will be better candidates, some will not be suitable at all, and that the best device has yet to be fabricated. Based on our current understanding, the ideal device would have low thresholds of adaptation (<0.2 V), on-state resistance of ∼100 kΩ or greater, high dynamic range, durability, the capability of incremental operation with very short pulse widths and long retention times of a week or more. However, even devices that deviate considerably from these parameters will be useful in more specific applications. As an example, short retention times on the order of seconds are perfectly compatible with combinatorial optimizers.

### AHaH Circuit Simulation

Circuit simulations were carried out by solving for the voltage at node y in each AHaH node (Figure 5) using Kirchhoff’s Current law (KCL) during the read phase followed by updating all memristor conductance values according to the generalized MSS model given the voltage drop across each memristor and the read period length. During the write phase, the memristor conductance values were individually updated according to the generalized MSS model given the voltage drop across each memristor and the write period length. The source code for the circuit is available in AHaH21Circuit.java. Parameters for operation of the circuit were set as follows: 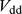 = 0.5 V, 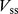 = −0.5 V, read period (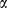) = 1 *μ*s, and write period (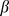) = 1 *μ*s. The number of input and bias memristors differed depending on the simulation task, as noted in each section below or in the source code.

### Spike Encoding

All machine learning applications built from AHaH nodes have one thing in common: the inputs to the AHaH nodes take as input a spike pattern. A spike pattern is a set of integers that specify which synapses in the AHaH node are coactive. In terms of a circuit, this is a description of what physical input lines are being driven by the driving voltage (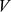). All other inputs remain floating (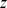). Any data source can be converted into a spike encoding with a spike encoder. As an example, the eye converts electromagnetic radiation into spikes, the ear converts sound waves into spikes, and the skin converts pressure into spikes. Each of these may be considered a spike encoder and each is optimized for a specific data source.

A simple example makes spike encoding for an AHaH node clear. Suppose a dataset is available where the colors of a person’s clothes are associated with the sex of the person. The entire dataset consists of several colors 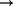 sex associations. For each person, the colors are mapped to an integer and added to a vector of variable length:
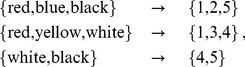
(28)where red maps to 1, blue maps to 2, yellow maps to 3, etc. The spike patterns for this dataset are then 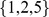, 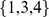, and 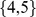. In order to accommodate the range of spikes, the AHaH nodes would require at least five inputs or a spike space of five.

In the case of real-value numbers, a simple recursive method for producing a spike encoding can also conveniently be realized through strictly anti-Hebbian learning via a binary decision tree with AHaH nodes at each tree node. Starting from the root node and proceeding to the leaf node, the input 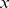 is summed with a bias 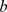, 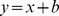. Depending on the sign of the result 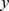, it is routed in one direction or another toward the leaf nodes. The bias is updated according to anti-Hebbian learning, the practical result being a subtraction of an adaptive average:
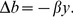
(29)


If we then assign a unique integer to each node in the decision tree, the path that was taken from the root to the leaf becomes the spike encoding. This process is an adaptive analog to digital conversion. The source code used to generate this spike encoding is in *AHaHA2D.java*. This adaptive binning procedure can be extended to sparse-spike encoded patterns if.
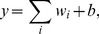
(30)where 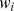 is sampled randomly from the set 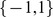 with equal frequency.

### Circuit and Functional Model Correspondence

We demonstrate that both the functional and circuit implementation of the AHaH node are equivalent and functioning correctly in order to establish a link between our benchmark results and the physical circuit. The source code for these experiments can be found in *AHaHRuleFunctionalApp.java* and *AHaHRuleCircuitApp.java* for both the functional and circuit form respectively. In both applications a four-input AHaH node receives the spike patterns from the set 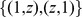, and the change in the synaptic weights is measured as a function of the output activation, y. Recall that we must encode the nonlinearly separable two-input channels into four-input linearly separable *spike logic* channels so that we can achieve all logic functions (XOR) directly with AHaH attractor states. For both the functional and circuit form of the AHaH node, a bias synapse is included in addition to the normal inputs.

In the derivation of the functional model, the assumption was made that the quantity 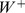 was constant (Equation 12). This enabled the treatment of the output voltage as a sum over the input and bias weights. This condition of conservation of adaptive resources is also required in the thermodynamic model (Equation 1). To demonstrate we have attained this conservation, the quantities 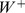 and 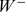 (Equations 12 and 14) are plotted for five different four-input AHaH nodes receiving the spike patterns from the set 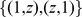 for 1100 time steps. The source code for this experiment is in *DifferentialWeightApp.java*.

### AHaH Logic

A two input AHaH node will receive three possible spike patterns 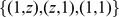 and converge to multiple attractor states. Each decision boundary plotted in Figure 2 represents a state and its anti-state (i.e. an AHaH bit), since two solutions exist for each stable decision boundary. The 6 possible states are labeled A, 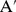, B, 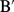, C, and 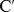. Fifty two-input AHaH nodes with Ag-chalcogenide memristors were simulated. All AHaH nodes were initialized with random weights picked from a Gaussian distribution with low weight saturation. That is, the memristors were initialized close to their minimally conductive states. Each node was given a stream of 500 inputs randomly picked with equal probability from the set 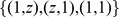. The source code for this experiment is in a file called *TwoInputAttractorsApp.java*, and there exists a functional form and a circuit form version to show correspondence between the two.

As stated earlier, the attractor states A, B, and C can be viewed as logic functions. It was earlier demonstrated how NAND gates can be used to make these attractor states computationally complete. It was also described how a spike encoding consisting of two input lines per channel can be used to achieve completeness directly with AHaH attractor states. To investigate this, 5000 AHaH nodes were initialized with random weights with zero mean. Each AHaH node was driven with 1000 spikes randomly selected from the set 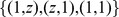. Finally, each AHaH node’s logic function was tested, and the distribution of logic functions was measured. The source code for this experiment is in *SpikeLogicStateOccupationFrequencyApp.java*, and there exists functional form and circuit form versions to show correspondence between the two.

To demonstrate that the attractor states and hence logic functions are stable over time, the above experiment can be repeated. However, the number of time steps can be significantly increased and the logic state of each AHaH node can be recorded at each time step. For this experiment, 100 AHaH nodes were randomly initialized, and their logic functions were tested over 50,000 time steps. The source code for this experiment is in *SpikeLogicFuntionVsTimeApp.java*, and there exists functional form and circuit form versions to show correspondence between the two.

### AHaH Clustering

Clustering is a method of knowledge discovery which automatically tries to find hidden structure in data in an unsupervised manner [101]. Centroid-based clustering methods like *k*-means [102] require that the user define the number of cluster centers ahead of time. Density-based methods can be used without predefining cluster centers, but can fail if the clusters are of various densities [103]. Methods such as OPTICS [104] address the problem of variable cluster densities, but introduce the problem that they expect some kind of density drop, which leads to arbitrary cluster borders. On datasets consisting of a mixture of known cluster distributions, density-based clustering algorithms are outperformed by distribution-based methods such as expectation maximization (EM) clustering [105]. However, EM clustering assumes that the data is a mixture of a known distribution and as such is not able to model density-based clusters. It is furthermore prone to over-fitting.

An AHaH node converges to attractor states that cleanly partition its input space by maximizing the margin between opposing data distributions. The set of AHaH attractor states are furthermore computationally complete. These two properties enable a sufficiently large collective of AHaH nodes to assign unique labels to unique input data distributions while maintaining a high level of tolerance to noise. If a collective of AHaH nodes are allowed to randomly fall into attractor states, the binary output vector is a label for the input feature. For example, a four node collective with outputs (0,0,0,1) would encode the output ‘0001’ and, if converted to base-10 integers, be assigned the cluster ID ‘1’. The collective node output (1,1,1,1) would encode the output ‘1111’ and be assigned the cluster ID ‘15’. Such a collective is called an AHaH clusterer.

The total number of possible output labels from the AHaH collective is 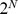, where 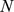 is the number of AHaH nodes in the collective. The collective may output the same label for different spike patterns if 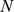 is small and/or the number of patterns, 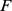, is high. However, as the number of AHaH nodes increases, the probability of this occurring drops exponentially. Under the assumption that all attractor states are equally likely, the probability that any two unique spike patterns, 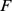, will be assigned the same binary label is:
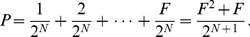
(31)


For example, given 64 spike patterns and 16 AHaH nodes, the probability of the collective assigning the same label is 3%. By increasing 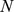 to 32, the probability falls to less than one in a million.

We developed a quantitative metric to characterize the performance of our AHaH clusterer. Given a unique spike pattern 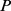 we would ideally like a unique label 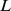 (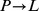). This is complicated by the presence of noise, occlusion, and non-stationary data or drift. Failure can occur in two ways. First, if the same underlying pattern is given more than one label, we may say that the AHaH clusterer is *diverging*. We measure the divergence, 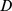, as the inverse of the average labels per pattern. Second, if two different patterns are given the same label, we may say that it is *converging*. We measure convergence, 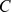, as the inverse of the average patterns per label.

Divergence and convergence may be combined to form a composite measure we call *vergence*, 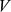:
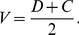
(32)


Perfect clustering extraction will occur with a vergence value of 1. The code used to encapsulate the vergence measurement can be found in *VergenceEvaluator.java*.

To investigate the AHaH clusterer’s performance as measured by our vergence metric, we swept the following parameters individually while holding the others constant: learning rate (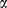, 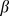), number of AHaH nodes, number of noise bits, spike pattern length, and number of spike patterns. The applications used to perform the sweeps can be found in the files *SweepLearningRateApp.java*, *SweepNumAhahNodesApp.java*, *SweepNumNoiseBitsVsSpikePatternLengthApp.java*, *SweepSpikePatternLengthApp.java*, and *SweepNumSpikePatternsApp.java*, respectively.

The number of inputs to the AHaH nodes making up the AHaH clusterer was 256. Synthetic spike patterns were created with a random spike pattern generator. Given a spike pattern length, the number of inputs available on the AHaH nodes and the number of unique spike patterns, a set of spike patterns was generated. Noise is generated by taking random input lines and activating them, or, if the input line is already active, deactivating it. The number of patterns that can be distinguished by the AHaH clusterer before vergence falls is a function of the input pattern sparsity, number of total patterns and the pattern noise. Both functional-based and circuit-based AHaH clusterers were investigated and showed good correspondence.

While the vergence experiments provide a quantitative measure of the characteristics of the AHaH clusterer, we also designed a program to qualitatively visualize the clustering capabilities. The basic idea is to create several spatial clusters in two-dimensional space and let the clusterer automatically determine the boundaries between clusters in an unsupervised manner. We used a *k*-nearest neighbor algorithm to translate the spatial location of cluster points into a spike representation, although other spike encoding methods are of course possible. The AHaH clusterer converges to attractor states that map spike patterns to integer, which is in turn mapped to a unique color. The visualizations give the observer a sense of how tolerant the AHaH clusterer is to variations in cluster type, size and temporal stability. The code for the clustering visualization is in *ClusteringApp.java*. There are several different visualizations including clusters of various sizes, arrangements, and numbers, both stationary and non-stationary.

### AHaH Classification

Linear classification is a useful tool used in the field of machine learning to characterize and apply labels to samples from datasets. State of the art approaches to classification include algorithms such as decision trees, random forests, support vector machines (SVM) and naïve Bayes [106]. These approaches are used in real world applications such as image recognition, data mining, spam filtering, voice recognition, and fraud detection. Our AHaH-based linear classifier is different from these techniques mainly in that it is not just another algorithm; it can be realized as a physically adaptive circuit. This presents several competitive advantages, the main one being that such a circuit would increase the speed and reduce power consumption dramatically while eliminating the problems associated with disk I/O bottlenecks experienced in large scale data mining applications [107].

The AHaH classifier consists of one or more AHaH nodes, each node assigned to a classification label and each operating the supervised form of the AHaH rule of Equation 17. In cases where a supervisory signal is not available, the unsupervised form of the rule (Equation 18) may be used. Larger magnitude AHaH node output activations are interpreted as a higher confidence. There are multiple ways to interpret the output of the classifier depending on the situation. First, one can order all AHaH node outputs and choose the most positive. This method is ideal when only one label per pattern is needed and an output must always be generated. Second, one can choose all AHaH node outputs that exceed a confidence threshold. This method can be used when multiple labels exist for each input pattern. Finally, only the most positive AHaH node output is chosen if it exceeds a threshold, otherwise nothing is returned. This method can be used when only one label per pattern is needed, but rejection of a pattern is allowed.

To compare the AHaH classifier to other state of the art classification algorithms, we chose four popular classifier benchmark data sets: the Breast Cancer Wisconsin (Original), Census Income, MNIST Handwritten Digits, and the Reuters-21578 data sets. The source code for these classification experiments is found in *BreastCancerFunctionalApp.java*, *CensusIncomeApp.java*, *MnistApp.java*, and *Reuters21578App.java*, respectively.

We scored the classifiers’ performance using standard classification metrics: precision, recall, F1, and accuracy. Information on these metrics and how they are used is widely available. The standard training and test sets were used for learning and testing respectively. More information about these benchmark datasets is widely available, and a large amount of classification algorithms have been benchmarked against them including SVM, naïve Bayes, and decision trees.

To further validate an AHaH classifier implemented with circuit AHaH nodes against functional AHaH nodes, we use the Breast Cancer Wisconsin (Original) benchmark dataset. This dataset is relatively small allowing the circuit level simulations to complete quickly. Each sample is either labeled *benign* or *malignant*. There were a total of 683 samples. The first 500 were designated as the training set and the last 183 as the test set. Our spike encoder for this data set produced a total of 70 unique spikes requiring 70 inputs for this particular classifier. The source code for the circuit form of the Breast Cancer Wisconsin experiment is in *BreastCancerCircuitApp.java*.

Continuous valued inputs were converted using the adaptive decision tree method of Equation 29. Text was converted to a bag-of-words representation where each unique word was representative of a unique spike. MNIST image data was first thresholded to produce a spike list of active pixels. The spike list in each 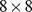 image patch was converted to a single spike via the method of Equation 30. The image patch was convolved and pooled over an 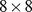 pixel region. The result of this procedure is a list of spikes with moderate translational invariance, which was fed to the AHaH classifier. The source code for this procedure is available in *MnistSpikeEncoder.java*.

The AHaH classifier is capable of unsupervised learning by evoking Equation 17. If no supervised labels are given but the classifier is able to output labels with high confidence, the output can be assumed to be correct and used as the supervised signal. The result is a continued convergence into the attractor state, which represent a point of maximal margin. This has application in any domain where large volumes of unlabeled data exist such as image recognition. By allowing the classifier to process these unlabeled examples, it can continue to improve its performance (bootstrap) without supervised labels.

To demonstrate this unsupervised learning capability we used the Reuters-21578 dataset. The entire training and test sets were lumped together and the classifier was given the first 25% inputs in a supervised manner. For the remaining 75% of the news articles, the classifier was run in an unsupervised manner. Only when the confidence was 1.0, which indicates high certainty of a correct answer, did the classifier use its own classification as a supervised training signal. The F1 score was recorded after each story for the following most frequent labels: *earn*, *acq*, *money-fx*, *grain*, *crude*, *trade*, *interest*, *ship*, *wheat*, and *corn*, a common label set used in many benchmarking exercises using this dataset. The source code for this experiment is in *Reuters21578SemiSupervisedApp.java*.

### AHaH Signal Prediction

Complex signal prediction involves using the prior history of a signal or group of signals to predict the future state of the signal. Signal prediction, also known as signal forecasting, is used in adaptive filters, resource planning and action selection. Some real world examples include production estimating, retail inventory planning, inflation prediction, insurance risk assessment, and weather forecasting. Current prediction algorithms include principle component analysis and regression and Kalman filtering [108], artificial neural networks [109] and Bayesian model averaging [110].

By posing signal prediction as a multi-label classification problem, complex signals can be learned and predicted using the AHaH classifier. As a simplified proof of concept exercise to demonstrate this, a complex temporal signal prediction experiment was designed. For each moment of time, the real-valued signal 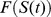 is converted into a sparse feature representation using the method of Equation 29. These features are buffered to form a feature set:
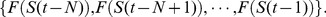
(33)


This feature set is then used to make predictions of the current feature 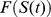, and the spikes of the current feature are used as supervised labels. After learning, the output prediction may be used in lieu of the actual input and run forward recursively in time. In this way, extended predictions about the future are possible. The source code for the experiment is available in *ComplexSignalPredictionApp.java*. The signal was generated from the summation of five sinusoidal signals with randomly chosen amplitudes, periods, and phases. The experiment ran for a total of 10,000 time steps. During the last 300 time steps, recursive prediction occurred.

### AHaH Motor Control

Motor control is the process by which sensory information about the world and the current state of the body is used to execute actions to generate movement. Stabilizing Hebbian feedback applied to an AHaH node can occur any time after the Anti-Hebbian read, which opens the interesting possibility of using AHaH nodes for reinforcement-based learning. Here we show that a small collective of AHaH nodes, an AHaH motor controller, can be used in autonomous robotic control. As a proof-of-concept experiment we use an AHaH motor controller to guide a multi-jointed robotic arm to a target based on a value signal or cost function.

**Figure 9:**
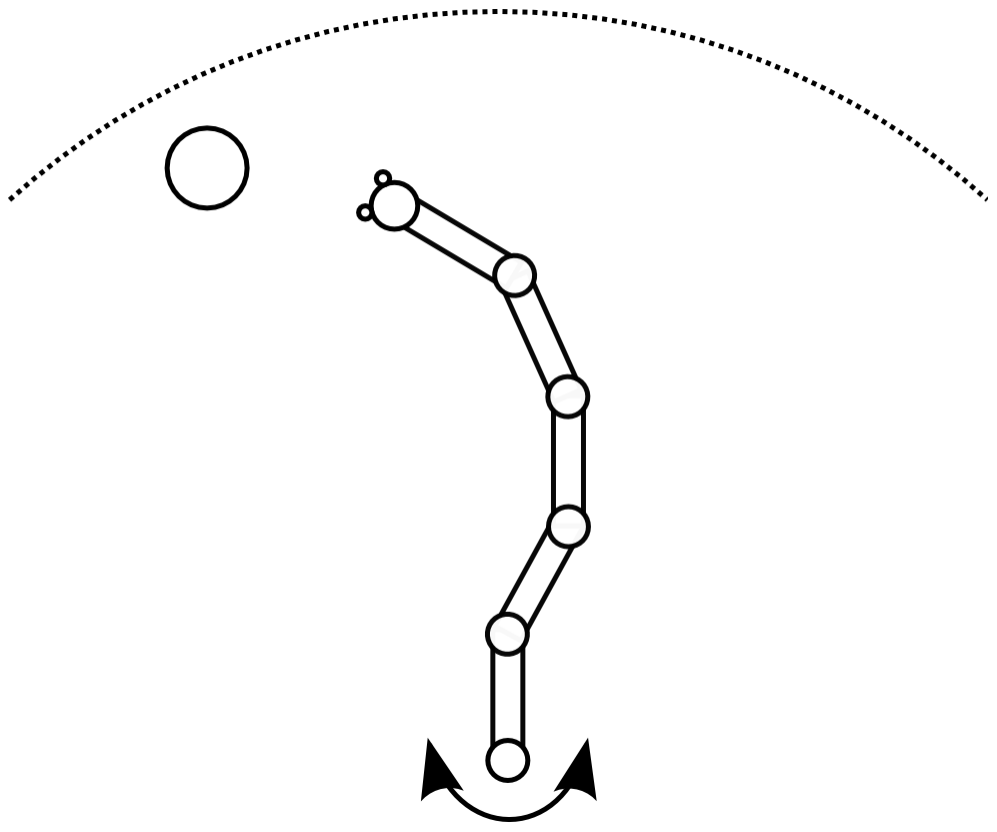
Unsupervised robotic arm challenge. The robotic arm challenge involves a multi-jointed robotic arm that moves to capture a target. Each joint on the arm has 360 degrees of rotation, and the base joint is anchored to the floor. Using only a value signal relating the distance from the head to the target and an AHaH motor controller taking as input sensory stimuli in a closed-loop configuration, the robotic arm autonomously learns to capture stationary and moving targets. New targets are dropped within the arm’s reach radius after each capture, and the number of discrete angular joint actuations required for each catch is recorded to asses capture efficiency.

A virtual environment in which an AHaH motor controller controls the angles of 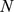 connected fixed length rods in order to make contact with a target was created as shown in Figure 9. The arm rests on a plane with its base anchored at the center, and all the joints have 360 degrees of freedom to rotate. New targets are dropped randomly within the robotic arm’s reach radius after it captures a target. The robotic arm virtual environment is part of an open source project called Proprioceptron, also available at http://xeiam.com. Proprioceptron builds upon a 3D gaming library and offers virtual worlds and challenges for testing motor control algorithms. The robotic arm challenge offers 5 levels of difficulty starting with stationary targets, increasing target lateral speed as the levels increase.

Sensors measure the relative joint angles of each segment of the robot arm as well as the distance from the target ball to each of two “eyes” located on the side of the arm’s “head”. Sensor measurements are converted into a sparse spiking representation using the method of Equation 29. A value signal is computed as the inverse distance of the head to the target:
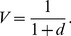
(34)


Opposing “muscles” actuate each joint. Each muscle is formed of many “fibers” and a single AHaH node controls each fiber. The number of discrete angular steps that move each joint, 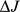, is given by:
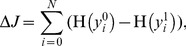
(35)where 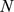 is the number of muscle fibers, 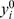 is the post-synaptic activation of the 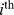 AHaH node controlling the 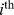 muscle fiber of the primary muscle, 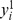 is the post-synaptic activation of the 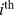 AHaH node controlling the 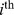 muscle fiber of the opposing muscle, and H is the Heaviside step function. The number of discrete angular steps moved in each joint at each time step is then given by the difference in these two values.

Given a movement we can say if a fiber (AHaH node) acted for or against it. We can further determine if the movement was good or bad by observing the change in the value signal. If, at a later time, the value increased after a movement, then each fiber responsible for the movement receive rewarding Hebbian feedback. Likewise, if the fiber acted in support of a movement and later the value signal dropped, then the fiber is denied a Hebbian update. As the duration of time between movement and reward increases, so does the difficulty of the problem since many movements can be taken during the interval. A reinforcement scheme can be implemented in a number of ways over a number of timescales and may even be combined. For example, we may integrate over a number of time scales to determine if the value increased or decreased.

Experimental observation led to constant values of 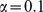 and 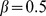 for the AHaH rule, although generally good performance was observed for a wide range of values. The choice of these parameters is influenced by the complexity of the problem and the need to learn complex compound sequences, as well as the duration between action (anti-Hebbian) and reward (Hebbian).

We measured the robotic arm’s efficiency in catching targets by summing the total number of discrete angular joint actuations from the time the target was placed until capture. As a control, the same challenge was carried out using a simple random actuator. The challenge was carried out for both AHaH-controlled and random-controlled robotic arm actuation for different robotic arm lengths ranging from 3 to 21 joints in increments of three. The total joint actuation is the average amount of discrete joint actuation over the 100 captured targets. The source code for this experiment is available in *RoboticArmApp.java*.

### AHaH Combinatorial Optimization

An AHaH node will descend into a probabilistic output state if the Hebbian feedback is withheld. As the magnitude of the synaptic weight falls closer to zero, the chance that state transitions will occur rises from 0% to 50%. This property can be exploited in probabilistic search and optimization tasks. Consider a combinatorial optimization task such as the traveling salesman problem where the city-to-city path is encoded as a binary vector 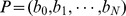. The space of all possible paths can be visualized as the leaves of a binary tree of depth 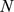. The act of constructing a path can be seen as a routing procedure traversing the tree from trunk to leaf. By allowing prior attempted solutions to modify the routing probabilities, an initial uniform routing distribution can collapse into a subspace of more optimal solutions.

This can be accomplished by utilizing an AHaH node with a single input as a node within a virtual routing tree. As a route progresses from the trunk to a leaf, each AHaH node is evaluated for its state and receives the anti-Hebbian update. Should the route result in a solution that is better than the average solution, all nodes along the routing path receive a Hebbian update. By repeating the procedure over and over again, a positive feedback loop is created such that more optimal routes result in higher route probabilities that, in turn, result in more optimal routes. The net effect is a collapse of the route probabilities from the trunk to the leaves as a path is locked in. The process is intuitively similar to the formation of a lighting strike searching for a path to ground and as such we call it a *strike search*.

To evaluate the AHaH combinatorial optimizer, we used the functional model (Equation 17), setting 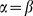 and making it a free parameter we call the *learning rate*, 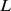:
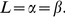
(36)


The experiment consists of 200 strike searches, where 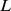 is set to a value chosen randomly from between 0.00015 and 0.0035 at the start of each trial. The noise variable, 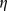, is picked from a random Gaussian distribution with zero mean and 0.025 variance. After every 10,000 solution attempts, branches with synaptic weight magnitudes less than 0.01 are pruned. A 64-city network is created where each city is directly connected to every other city, and the city coordinates are picked from a random Gaussian distribution with zero mean and a variance of one. The city path is encoded as a bit sequence such that the first city is encoded with 6 bits, and each successive city with only as many bits needed to resolve the remaining cities such that the second-to-last city requires one bit. The value of the solution is the path distance, which we are attempting to minimize. The strike process is terminated when the same solution is generated 5 successive times, indicating convergence. A random search is used as a control, where each new solution attempt is picked from a uniform random distribution. The code for this experiment is in *StrikeSearchApp.java*.

## Results and Discussion

### AHaH Rule

**Figure 10:**
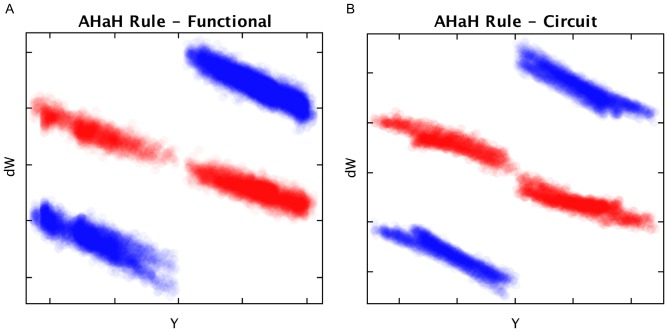
The AHaH rule reconstructed from simulations. Each data point represents the change in a synaptic weight as a function of AHaH node activation, y. Blue data points correspond to input synapses and red data points to bias inputs. There is good congruence between the A) functional and B) circuit implementations of the AHaH rule.

The AHaH rule reconstructions for the functional and circuit forms of the AHaH node are shown in Figures 10A and 10B respectively. In both cases, the AHaH rule is clearly represented and there is congruence between both forms. However, Figure 10B hides complexity in the circuit that arises from the differential aspect of the weights and their limited dynamic range. Because of this, depending on the saturation state of a weight, the form of weight update may change over time. The AHaH rule reconstruction of Figure 10B is thus for a specific weight initialization for a specific time interval.

**Figure 11:**
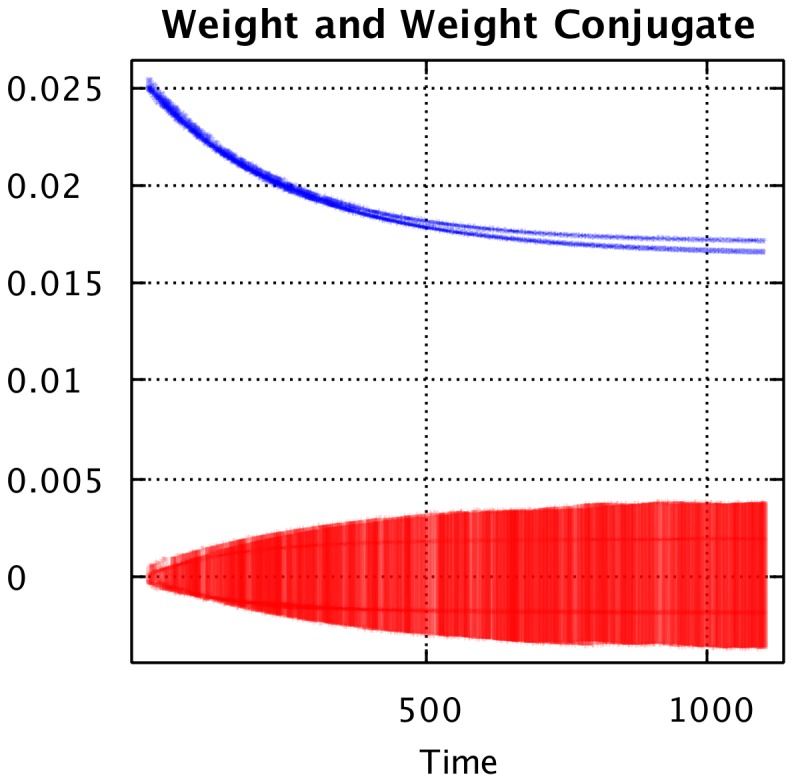
Justification of constant weight conjugate. Multiple AHaH nodes receive spike patterns from the set 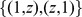 while the weight and weight conjugate is measured. Blue = weight conjugate (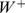), Red = weight (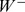). The quantity 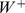 has a much lower variance than the quantity 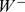 over multiple trials, justifying the assumption that 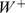 is a constant factor.

As part of our functional model derivation (Equation 12) and the connection to thermodynamics (Equation 1) we require that the quantity 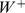 remains constant. As can be seen in Figure 11, the quantity 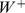 does indeed asymptote and remains constant.

### AHaH Logic

**Figure 12:**
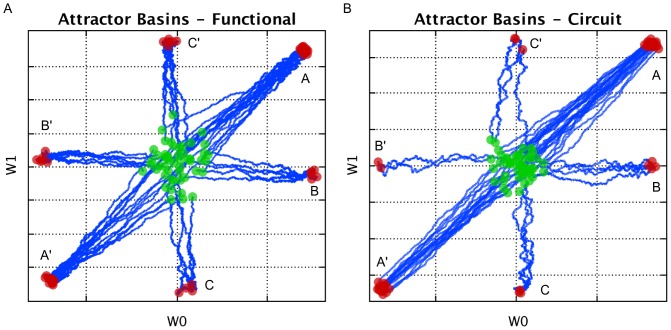
Attractor states of a two-input AHaH node under the three-pattern input. The AHaH rule naturally forms decision boundaries that maximize the margin between data distributions. Weight space plots show the initial weight coordinate (green circle), the final weight coordinate (red circle) and the path between (blue line). Evolution of weights from a random normal initialization to attractor basins can be clearly seen for both the functional model (A) and circuit model (B).

The 2-input AHaH node receiving 500 consecutive inputs randomly chosen from the set 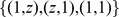 at 50 different initial synaptic weights evolves into one of the six attractor basins as shown in Figure 12. Labels A, 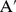, B, 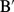, C, and 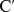 indicate the attractor basins in these weight-space plots and correspond to the equivalent decision boundaries shown in Figure 2. The same experiment was performed with the functional form and the circuit form of the AHaH node (Figure 12A and 12B respectively) and close correspondence can be seen.

**Figure 13:**
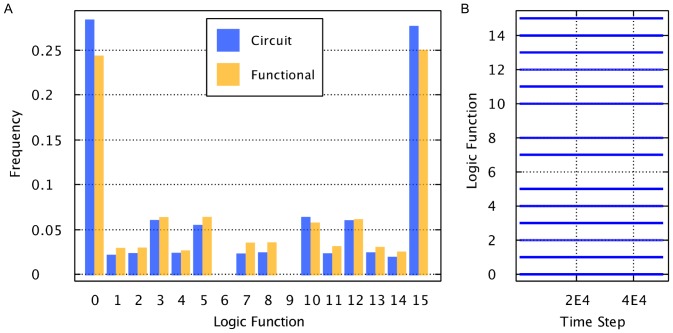
AHaH attractor states as logic functions. A) Logic state occupation frequency after 5000 time steps for both functional model and circuit model. All logic functions can be attained directly from attractor states except for XOR functions, which can be attained via multi-stage circuits. B) The logic functions are stable over time for both functional model and circuit model, indicating stable attractor dynamics.

After being initialized with random synaptic weights, the occupation of logic states of AHaH nodes receiving the spike logic patterns of Table 1 are shown in Figure 13 for both functional and circuit models. Each logic function was assigned a unique integer value as in Table 4. Experimental results show congruence between the functional form and circuit form simulations. All linear functions are represented by distinct AHaH attractor states. Absent are the expected nonlinear XOR functions 6 and 9. These functions are possible through combinations of other logic functions, meaning a multi-stage AHaH node network is capable of achieving any logic function. Since any algorithm or program can be reduced to successive utilizations of logic gates, the attractor states of AHaH nodes support universal computation. Logic functions remain stable over time, as indicated by Figure 13B.

**Table 4:** Logic functions.

SP⇓, LF⇒	15	14	13	12	11	10	9	8	7	6	5	4	3	2	1	0
(*z*, 1, *z*, 1)	1	1	1	1	1	1	1	1	0	0	0	0	0	0	0	0
(*z*, 1, 1, *z*)	1	1	1	1	0	0	0	0	1	1	1	1	0	0	0	0
(1, *z*, *z*, 1)	1	1	0	0	1	1	0	0	1	1	0	0	1	1	0	0
(1, *z*, 1, *z*)	1	0	1	0	1	0	1	0	1	0	1	0	1	0	1	0

The table defines all 16 possible logic functions (LF) for the four spike encoded input patterns (SP).

Logic functions 0 and 15 represent the null state and their occupation is inhibited through the action of the bias. By increasing the number of bias inputs from 1 to 3 we can collapse the stable attractor logic states down to 3, 5, 10 and 12. These functions represent the pure independent component states and act to pass or invert each of the two input channels. Although these states are not computationally complete, they can be made so via the use of NAND gates as we discussed in the theory section. The advantage of using states 3, 5, 10 and 12 is that they are very stable. The disadvantage is that we must now rely on external circuitry (i.e. NAND gates) to achieve computational universality.

### AHaH Clustering

The AHaH clusterer parameter sweep experiment results are summarized in Table 5. While setting the free parameters at their default values and sweeping the parameter under investigation, the range of that parameter that resulted in a vergence value greater than 0.90 was determined. The performance of the AHaH clusterer proved to be robust to input pattern noise. For example, the clusterer can achieve perfect performance with up to 18% noise under a 100% pattern load. A full pattern load occurs when the number of patterns (16) multiplied by the pattern size (16) is equal to the total number of input lines (256 in this case). The clusterer can achieve greater than 90% vergence with up to 44% noise, meaning 7 of the 16 spike input pattern’s bits are reassigned random values.

**Table 5:** AHaH clusterer sweep results.

	Learning Rate	Number ofAHaH nodes	Number ofNoise Bits	Spike PatternLength	Number ofSpike Patterns
Default Value	0.0005	20	3	16	16
Range	.0002–.0012	>7	< = 7	< = 36	< = 28

While sweeping each parameter of the AHaH clusterer and holding the others constant at their default values, the reported range is where the vergence remained greater than 90%.

**Figure 14:**
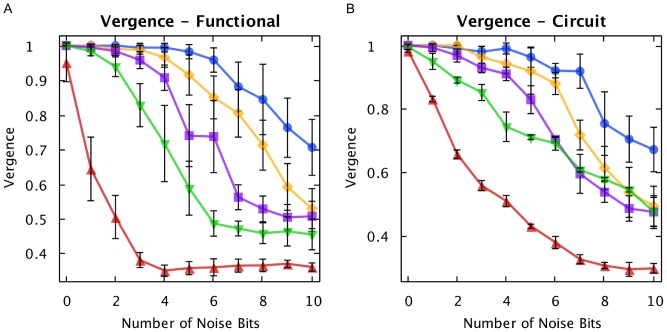
AHaH clusterer. Functional (A) and circuit (B) simulation results of an AHaH clusterer formed of twenty AHaH nodes. Spike patterns were encoded over 16 active input lines from a total spike space of 256. The number of noise bits was swept from 1 (6.25%) to 10 (62.5%) while the vergence was measured. The performance is a function of the total number of spike patterns. Blue = 16 (100% load), Orange = 20 (125% load), Purple = 24 (150% load), Green = 32 (200% load), Red = 64 (400% load).

The results shown in Figure 14 illustrate that the performance as measured by vergence degrades as the number of spike patterns increase. This result is explained by the fact that AHaH plasticity is acting to maximize the margin between data distributions or patterns. As the number of patterns increases, the margin must decrease and hence becomes more susceptible to noise. For example, under a 200% pattern load (32 patterns), vergence falls below 90% after 12.5% noise (2 noise bits). Comparing Figure 14A and 14B further demonstrates that circuit and functional models produce similar results. Without noise, the clusterer has impressive capacity and can reliably assign labels to spike patterns with load factors that exceed 400%.

**Figure 15:**
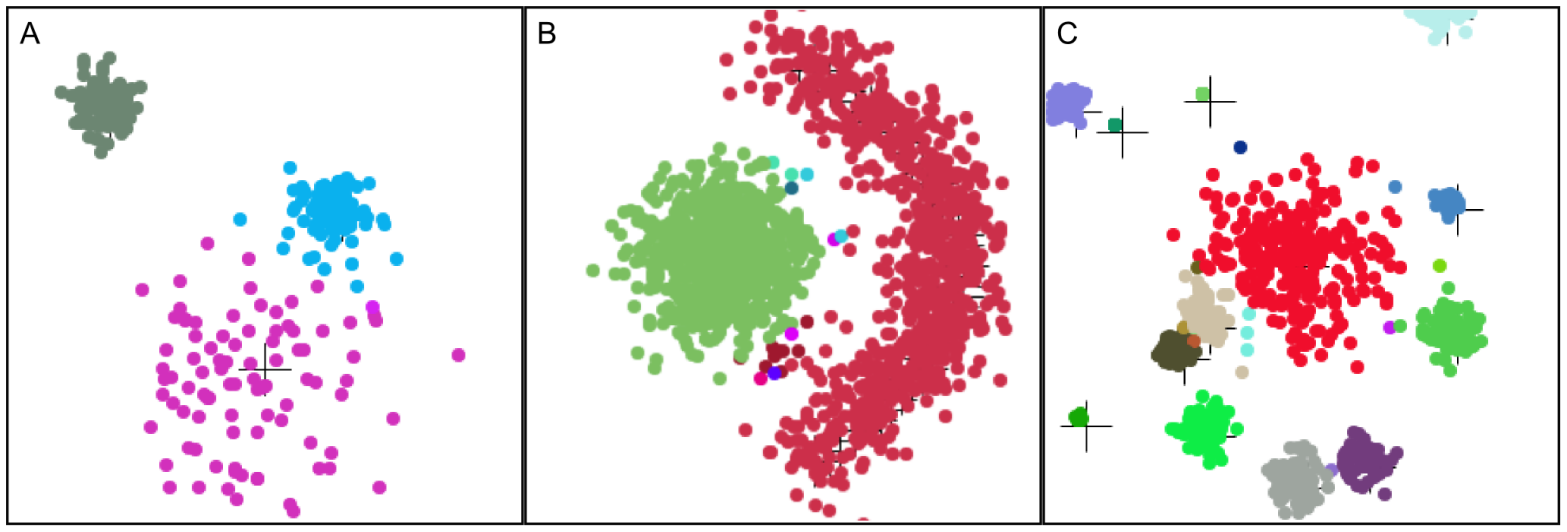
Two-dimensional spatial clustering demonstrations. The AHaH clusterer performs well across a wide range of different 2D spatial cluster types, all without predefining the number of clusters or the expected cluster types. A) Gaussian B) non-Gaussian C) random Gaussian size and placement.

Figure 15 shows screen shots of three different two-dimensional clustering visualizations. The AHaH clusterer performs well for clusters of various sizes and numbers as well as non-Gaussian clusters even though it does not need to know the number of clusters ahead of time or the expected cluster forms. Videos of similar clustering tasks shown in Figure 15 can be viewed in the online Supporting Information section (Videos S1– S3). Video S4 shows a two-dimensional clustering visualization with moving clusters.

The results show that the AHaH clusterer is able to handle a spectrum of cluster types. We demonstrate the ability to detect Gaussian and non-Gaussian clusters, clusters of non-equal size, as well as non-stationary clusters. Whereas other methods have intrinsic failure modes for certain types of clusters, our method can apparently handle a wide range of cluster types. Although more work must be done to fully compare our methods to existing clustering methods, our results thus far indicate that our method offers a genuinely new clustering mechanism with a number of distinct advantages. The most significant advantage is that we can implement the AHaH clusterer in physically adaptive AHaH circuits. In other words, clustering can now become an adaptive hardware resource.

### AHaH Classification

Our AHaH classifier benchmark scores for the Breast Cancer Wisconsin (Original), Census Income, MNIST Handwritten Digits, and the Reuters-21578 data sets are shown in Table 6 along with results from other published studies using their respective classification methods. Our results compare well to published benchmarks and consistently match or exceed SVM performance. This is surprising given the simplicity of the approach, which amounts to simple sparse spike encoding followed by classification with independent AHaH nodes.

**Table 6:** Benchmark classification results.

Breast Cancer Wisconsin (Original)		Census Income		MNIST Handwritten Digits		Reuters-21578	
AHaH	.997	AHaH	.853	AHaH	.98–.99	AHaH	.92
RS-SVM [115]	1.0	NBTree [116]	.86	deep convex net [117]	.992	SVM [118]	.864
SVM [119]	.972	naïve-Bayes [116]	.84	large conv. net [120]	.991	C4.5 [118]	.794
C4.5 [121]	.9474	C4.5 [116]	.858	polynomial SVM [42]	.986	naïve-Bayes [118]	.72

AHaH classifier classification scores for the Breast Cancer, Census Income, MNIST Handwritten Digits and Reuters-21578 classification benchmark datasets. The AHaH classifier results compare favorably with other methods. Higher scores on the MNIST dataset are possible by increasing the resolution of the spike encoding.

In comparing our MNIST results with other methods, it is important to account for data preprocessing and artificial inflation of the training data set through transformations of training samples. We do not inflate the training set; our results are achievable with only one online training epoch. Both the training and test are completed on a standard desktop computer processor in a few minutes to less than an hour, depending on the resolution of the spike encoding. The current state of the art achieves a recognition rate of 99.65% and “took a few days” to train on a desktop computer with GPU acceleration [111]. Another study determined that human performance on this task is 97.27% [112].

The Reuters-21578, Census Income and Breast Cancer datasets cover a range of data types from strings to integers to continuous real-valued signals. The Census Income dataset furthermore contains mixed data types as well as exemplars with missing attributes. In all cases the AHaH classifier combined with the simple spike encoder of Equation 29 matched or exceeded state of the art classifiers. This is significant primarily for the reason that both spike encoding and classification functions can be attained via AHaH learning and support the idea that a generic adaptive learning hardware resource is possible.

**Figure 16:**
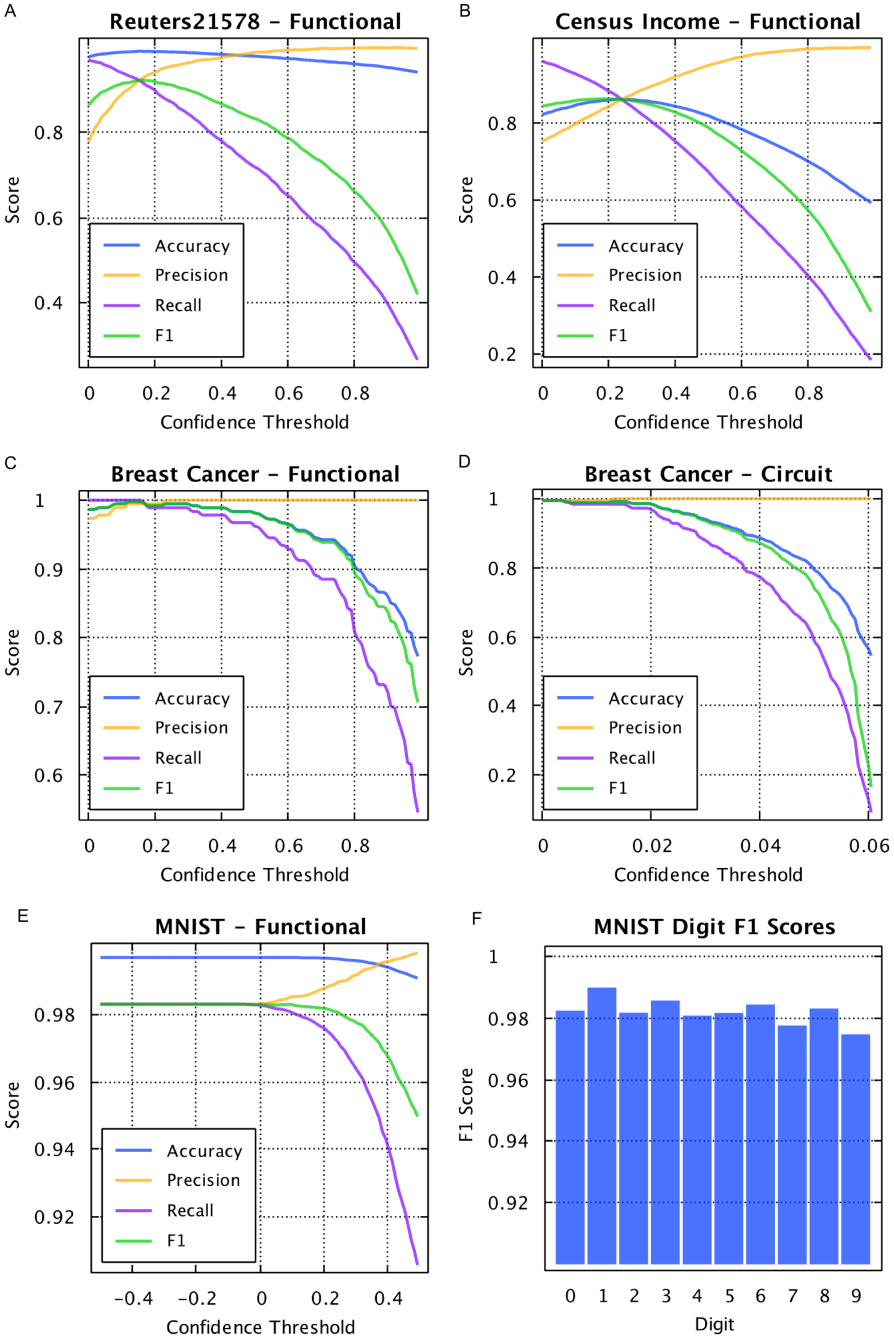
Classification benchmarks results. A) Reuters-21578. Using the top ten most frequent labels associated with the news articles in the Reuters-21578 data set, the AHaH classifier’s accuracy, precision, recall, and F1 score was determined as a function of its confidence threshold. As the confidence threshold increases, the precision increases while recall drops. An optimal confidence threshold can be chosen depending on the desired results and can be dynamically changed. The peak F1 score is 0.92. B) Census Income. The peak F1 score is 0.853 C) Breast Cancer. The peak F1 score is 0.997. D) Breast Cancer repeated but using the circuit model rather than the functional model. The peak F1 score and the shape of the curves are similar to functional model results. E) MNIST. The peak F1 score is 0.98–.99, depending on the resolution of the spike encoding. F) The individual F1 classification scores of the hand written digits.

Figure 16 provides a more detailed look at the individual classification experiments. Typical for all benchmark data sets, as the confidence threshold of the AHaH classifier is increased, the precision increases while recall drops (Figure 16A and 16B). In other words, the classifier makes fewer mistakes at the expense of not being able to answer some queries. The circuit-level simulation using the Ag-chalcogenide device model yielded a classification score as a function of confidence threshold similar to the functional simulations as shown in Figures 16C and 16D respectively. Similar circuit-level simulation results were obtained using the AIST and WO*_x_* device models. The results of the MNIST experiment are shown in Figures 16E and 16F. While Figure 16E shows the average over all digits, Figure 16F shows the scores of the individual digits.

**Figure 17:**
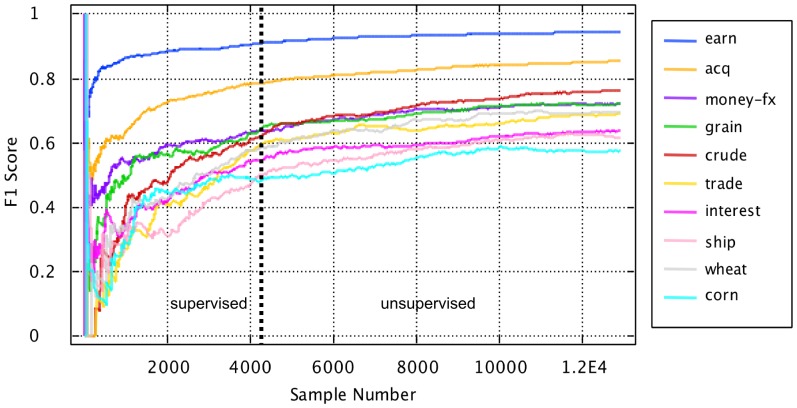
Semi-supervised operation of the AHaH classifier. For the first 30% of samples from the Reuters-21578 data set, the AHaH classifier was operated in supervised mode followed by operation in unsupervised mode for the remaining samples. A confidence threshold of 1.0 was set for unsupervised application of a learn signal. The F1 score for the top ten most frequently occurring labels in the Reuters-21578 data set were tracked. These results show that the AHaH classifier is capable of continuously improving its performance without supervised feedback.

Using the confidence threshold as a guide, the AHaH classifier can also be used in a semi-supervised mode. Starting in supervised mode and learning over a range of training data, the classifier can then switch to unsupervised mode. In unsupervised mode we may activate Hebbian learning if the confidence exceeds a value. Results are shown in Figure 17, which shows continued improved F1 score without supervision.

Results to date indicate that the AHaH classifier is an efficient incremental optimal linear classifier. The AHaH classifier displays a range of desirable classifier characteristics hinting that it may be an ideal general classifier capable of handling a wide range of classification applications. The classifier can learn online in a feed-forward manner. This is important for large data sets and applications that require constant adaptation such as prediction, anomaly detection and motor control. The classifier can associate an unlimited number of labels to a pattern, where the addition of a label is simply the addition of another AHaH node. By allowing the classifier to process unlabeled data it can improve over time. This has practical implications in any situation where substantial quantities of unlabeled data exist. Through the use of spike encoders, the classifier can handle mixed data types such as discrete or continuous numbers and strings. The classifier tolerates missing values, noise, and irrelevant attributes and is computationally efficient. The most significant advantage, however, is that the circuit can be mapped to physically adaptive hardware. Optimal incremental classification can now become a hardware resource.

### AHaH Signal Prediction

**Figure 18:**
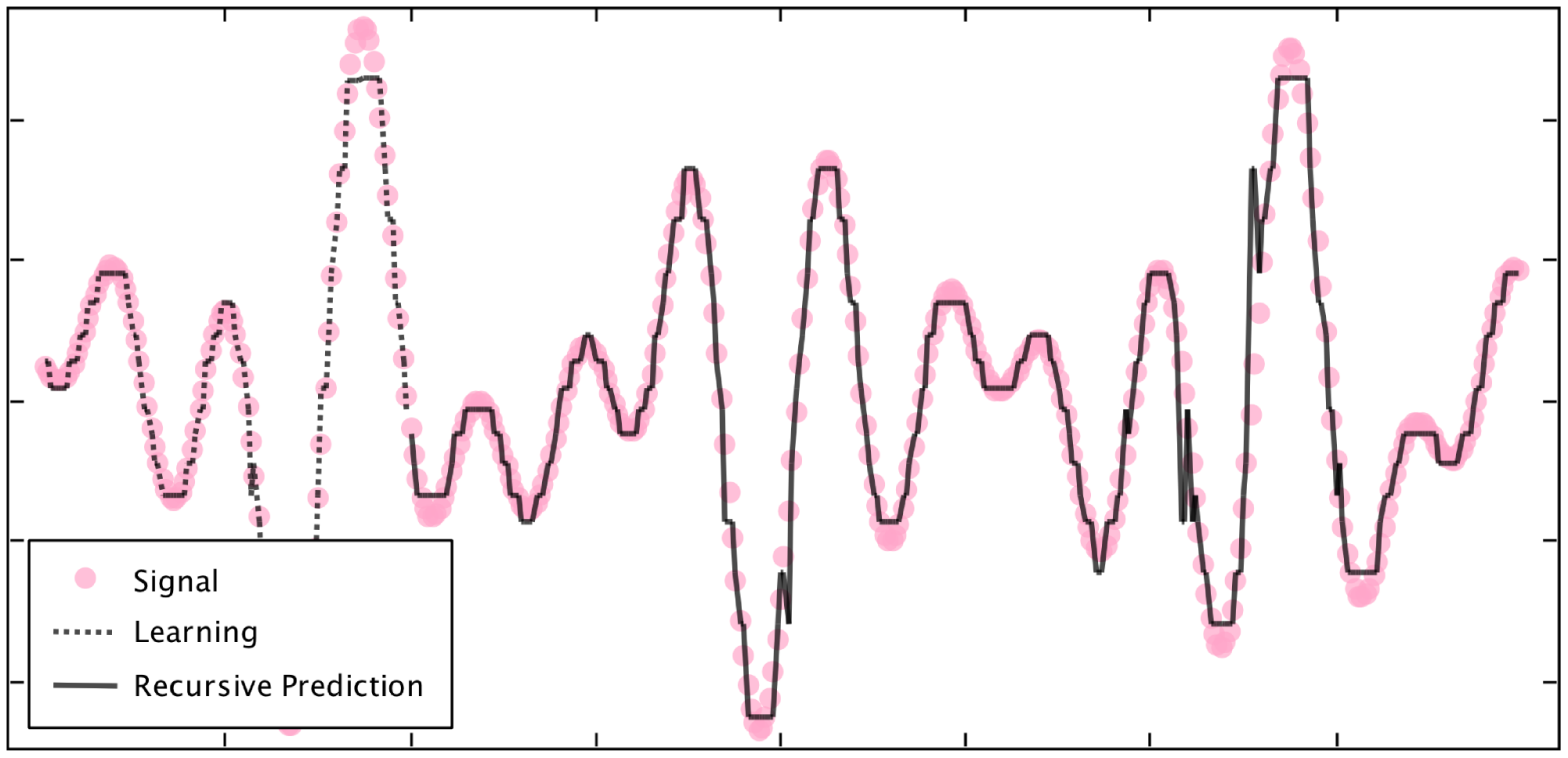
Complex signal prediction with the AHaH classifier. By posing prediction as a multi-label classification problem, the AHaH classifier can learn complex temporal waveforms and make extended predictions via recursion. Here, the temporal signal (dots) is a summation of five sinusoidal signals with randomly chosen amplitudes, periods, and phases. The classifier is trained for 10,000 time steps (last 100 steps shown, dotted line) and then tested for 300 time steps (solid line).

The results of the temporal signal prediction experiment are shown in Figure 18. The solid line drawn on top of the true signal represents the predictor’s accurate prediction of the true complex waveform after a period of supervised learning (mostly not shown). One advantage of the recursive prediction is that the forward-looking time window can be dynamically chosen. Although the predictor was trained to predict only the next time step, the recursive prediction can be carried forward to the desired point in the future for which the prediction should be made, which was 300 time steps in this example. At some point forward the prediction will degrade if the signal is not deterministic and cyclical. Not all applications require the recursive prediction, and a simpler statically set forward-looking time window could be set.

While this temporal signal prediction demonstration is not by any means an exhaustive comparison of AHaH signal prediction to other forecasting algorithms, it demonstrates the utility and flexibility of the AHaH classifier and provides the first glimpse of using AHaH nodes in the large application space of signal forecasting. These results also shed light on how AHaH node supervisory signals could be generated in a completely self-organizing system with zero human intervention. Time is the supervisor and prediction is the Hebbian reward. From the practical perspective, prediction provides the ability to prepare or optimize for the future. It also provides the ability to detect when a system is changing. If a prediction fails to meet with reality, an anomaly has occurred.

### AHaH Motor Control

**Figure 19:**
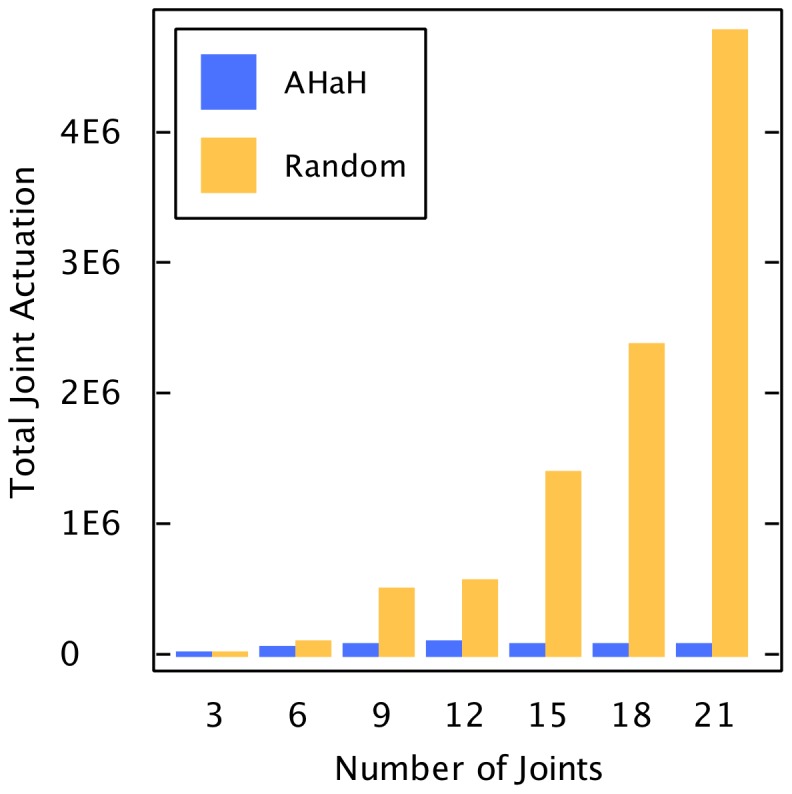
Unsupervised robotic arm challenge. The average total joint actuation required for the robot arm to capture the target remains constant as the number of arm joints increases for actuation using the AHaH motor controller. For random actuation, the required actuation grows exponentially.

The results of the motorized robotic arm experiment are shown in Figure 19. The performance of the AHaH-guided robotic arm is compared with a random-guided robotic arm by measuring the average total joint actuation needed to capture 100 moving targets. The results demonstrate that the collective of AHaH nodes are performing a gradient descent of the value function and can rapidly guide the arm to its target, independent of the number of joints. Videos of AHaH-controlled 3-, 6-, 9-, 12-, and 15-joint robotic arms performing the capture challenge can be viewed in the online Supporting Information section (Videos S5–S9).

Our results show that populations of independent AHaH nodes can effectively control multiple degrees of freedom so as to ascend (or descend) a value function. This process is spontaneous and results from the emergent behavior of many AHaH nodes acting as *self configuring classifiers* competing for Hebbian reward. Real world applications of this effect could of course include actuation of robotic appendages as well as autonomous controllers. Our results are significant primarily because the controller can be reduced to physically adaptive circuits and hence can be made to consume very little power and space, an important consideration in mobile platforms.

### AHaH Combinatorial Optimization

**Figure 20:**
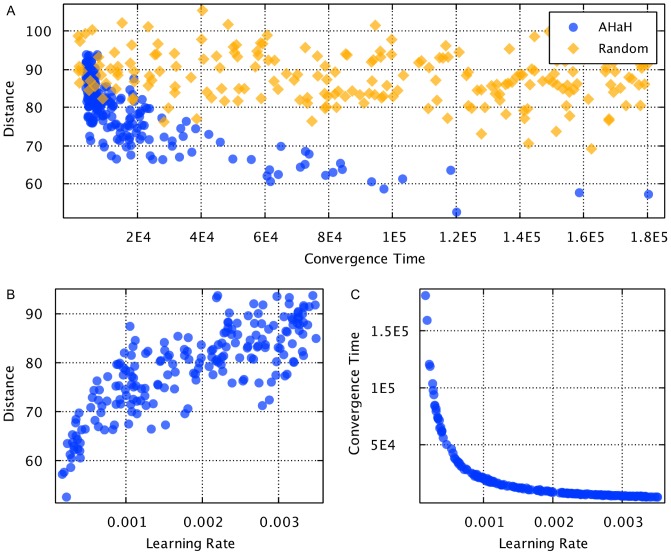
64-city traveling salesman experiment. By using single-input AHaH nodes as nodes in a routing tree to perform a strike search, combinatorial optimization problems such as the traveling salesman problem can be solved. Adjusting the learning rate can control the speed and quality of the solution. A) The distance between the 64 cities versus the convergences time for the AHaH-based and random-based strike search. B) Lower learning rates lead to better solutions. C) Higher learning rates decrease convergence time.

The results of the traveling salesman problem experiment are shown in Figure 20. Our experiment demonstrates that an AHaH combinatorial optimizer performing a strike search can outperform a strike search backed by a random path chooser (Figure 20A). This result shows that the strike is performing a directed search as expected. Trials with higher convergence times resulted from cases where the optimizer was given a relatively lower learning rate. Recall, a lower learning rate allows for a finer-grained search resulting in the longer convergence times. Figure 20B shows the relationship between the learning rate and the solution value (distance), while Figure 20C shows the relationship between the learning rate and the convergence time. Lowering the learning rate causes more evidence to be accumulated before positive-feedback forces selection and the solution proceeds from the trunk to leaf node, one bit at a time.

A strike evolves in time as bits are sequentially locked in via the positive feedback selection mechanism after a period of evidence accumulation. The lower the learning rate, the more evidence is accumulated before a path is locked in. In this way, a strike search appears to be a relatively generic method to accelerate the search for a procedure. Using the traveling salesman problem as an example, we could just as easily encode the strike path as a relative procedure for re-ordering a list of cities rather than an absolute ordering. For example, we could swap the cities at indices A and B, then swap the cities at indices C and D, and so on. Furthermore, we could utilize the strike procedure in a recursive manner. In the case of the traveling salesman problem we could assign lower-level strikes to find optimal sub-paths and higher-order strikes to assemble larger paths from the sub-paths. Most generally, if (1) a problem can be represented as a bit configuration and (2) the configuration can be assigned a value in an efficient manner, then a strike can be used as an adaptive learning hardware resource for optimization tasks. The ability to change the convergence times allows dynamic choices to be made in the time available.

### Synaptic Power Consumption

Both static and dynamic power consumption pathways must be considered when calculating the energy dissipation of neuromorphic chips containing AHaH circuit architecture. The static power component is dominated by the current flowing through the AHaH node synapse arrays during the read and write phases. The dynamic power component is dominated by the charging and discharging of the capacitive components of the circuitry. This capacitance includes parasitics from circuit elements and interconnect wires. Industry best practices can optimize dynamic power consumption. Here we focus on an estimation of static power consumption. Note that by not including the dynamic power consumption in this estimation, these values represent only a lower bounds on the synaptic power consumption of a neuromorphic chip. Dynamic power consumption, which is heavily dependent on chip design and architecture may have a significant power contribution. Recall that one of the major motivations of AHaH computing is the elimination of the von Neumann bottleneck for machine learning applications. Considering static and dynamic power consumption together with the elimination of this bottleneck, the net gain in power efficiency compared to modern digital electronics will most likely increase.

Static power dissipation of a single AHaH node is equal to 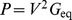, where 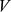 is the voltage drop across the memristor pairs and 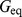 is the equivalent conductance of the combined active input and bias memristor pairs in Figure 5. Since each synapse only dissipates energy when it is active (it remains floating otherwise), and since only a small number of synapses are active at any given time (given the sparse spike encoding), the current flowing through the AHaH node during the read and write phases is very low. The total dissipative energy per synaptic event is.
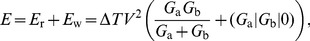
(37)where 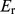 and 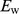 are the energy of the read and write phases, respectively, 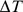 is the pulse width of the read and write phases and 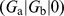 denotes the three possible outcomes 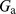, 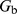 or 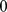. That is, the synapse could select the *A Path*, the *B Path* or else feedback is withheld. Utilizing Equation 1, it is straightforward to show the conductance under maximum power dissipation for each condition, as shown in Table 7.

**Table 7:** Maximum power and corresponding synaptic weights.

Condition	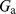	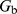	Maximum Power
Path A Selected	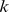	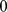	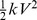
Path B Selected	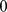	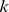	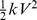
No Feedback	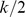	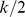	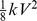

The maximum power dissipation of a differential synaptic weight changes depending on whether feedback is present or not. In the absence of feedback, the power is maximized when the conductance of each path is the same and the output descends into randomness. When feedback is present the synapse may converge to one of two possible configurations, and the power dissipation increases by a factor of four.

From Equation 37 it is clear that lower operating voltages, shorter pulse widths, and lower conductance memristors will reduce static power consumption. Developing an ideal candidate memristor for AHaH computing will play an important role in static power reduction. Given an operating resistance of the Ag-chalcogenide memristor of 250Ω, a pulse with of 10 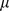
s and a voltage of 1 V, the estimated per-synapse static energy consumption is 40 nJ. Several synapse-like memristors achieving ultra-low power pulsed synaptic updates have recently been reported [68], [70], [113]. The energy consumption per synapse using roughly average voltage, pulse width, and resistance values taken from the pulse-driven memristive behavior plots in the three references results in a calculated energy consumption per synapse per update of ∼12 pJ, ∼67.5 pJ, and ∼56 pJ respectively. Kuzum et al. [113] claim that scaling trends project energy consumption for electronic synapses down to 2 pJ. As an example, a 100 kΩ device driven with 1 V, 100 ns pulse widths would consume 1.5 pJ of static energy per synapse. Such devices will play a significant role in reaching biological efficiency.

In all applications, the spike encoding plays an important role in reducing the number of spikes and hence the power consumption. Table 8 tabulates the coactive spikes, the spike space, and the number of AHaH nodes used for most of the demonstration applications and benchmarks in this paper. Different applications require different configurations; some will have few AHaH nodes and a large spike space while others may have many AHaH nodes and few inputs.

**Table 8:** Application spike sparsity and AHaH node count.

Application	CoactiveSpikes	SpikeSpace	Sparsity	AHaH NodeCount
Breast Cancer	31	70	0.44	2
Census Income	63	∼1800	∼0.035	2
MNIST	∼1000	∼27,500	∼0.036	10
Reuters 21578	∼100	∼46,000	∼0.002	119
Robotic Arm	92	341	0.27	345
Comb. Opt.	1	1	n/a	∼600,000
Clusterer	16	256	0.0625	20
Prediction	300	9600	0.031	32

The applications and benchmarks presented in this paper to demonstrate various machine learning tasks using AHaH plasticity require different AHaH node configurations depending on the type of data being processed and what the desired result is. The sparsity is a function of the incoming data and is defined as the number of coactive spikes divided by the total spike space.

## Limitations of the Study, Open Questions, and Future Work

We have attempted to connect a low-level general statistical model of collections of metastable switches with dissipative attractor-based computation and machine learning in a physically realizable circuit. Our aim is to provide a road map for others to follow so that we may all explore and exploit this interesting and potentially useful form of computing. Our ultimate goal is to provide a physical adaptive learning hardware resource (the AHaH circuit) in much the same way as modern RAM memory provides a memory resource to computing systems. However, only when we have investigated the circuit and functional models and have demonstrated real world utility is it necessary to move toward simulation of nonideal circuits effects, such as parasitic impedances, signal delays, settling times and variations in memristor properties. These details are certainly required for the eventual construction of a neural processing unit (NPU) but to include them in this paper would obfuscate our core message that “a new type of computing is possible that appears to offer a solution of general machine learning”.

Our demonstrations of utility include results across the field of machine learning, from clustering and classification to prediction, control and combinatorial optimization. Given the intended broad scope of this paper it was not possible to provide much elaboration on some of our results, comparison with many other methods, nor discuss the implications. For this reason we have open-sourced all code used to generate the results of this paper. We encourage the reader to investigate our methods carefully and come to their own conclusions.

Although it was important to develop specific techniques to address the broad capabilities we have demonstrated, we wish to convey the idea that the AHaH node is a building block from which many higher-order adaptive algorithms may be built including many we have not yet conceived of. As an example consider our results with the AHaH motor controller and AHaH classifier. By using the classifier’s confidence estimation as the value function for the AHaH motor controller, which in turn controls the viewing position, angle and rotation of an “eye”, it should be possible to spontaneously control the gaze of a vision system to find and center previously trained objects. Alternately, by pairing the AHaH signal prediction with the AHaH combinatorial optimizer, it should be possible learn to predict a reward signal while simultaneously optimizing actions to attain reward. We can infer from our results that other capabilities are possible. Anomaly detection, for example, is the inverse of prediction. If a prediction can be made about a temporally dynamic signal, then an anomaly signal can be generated should predictions fail to match with reality. Tracking of non-stationary statistics is also a natural by-product of the attractor nature of the AHaH rule, and was slightly touched upon in the 2D clustering videos, Video S4 in particular. Attractor points of the AHaH rule are created by the data structure. It follows logically that these same states will shift as the structure of the information changes. It also follows that a system built of components locked in attractor states will spontaneously heal if damaged [43], [44]. This property could provide new developments in fault-tolerant electronics.

## Conclusions

We have introduced the concept of AHaH computing. We have shown how the simple process of particles dissipating into containers through adaptive channels competing for conduction resources leads to AHaH plasticity. We have shown that memristive devices can arise from metastable switches, how differential synaptic weights may be built of two or more memristors, and how an AHaH node may be built of arrays of synapses. A simple read and write cycle driving an AHaH circuit results in physical devices implementing AHaH plasticity. We have demonstrated that the attractor states of the AHaH rule can configure computationally complete logic functions, and have shown their use in supervised and unsupervised classification, clustering, complex signal prediction, unsupervised robotic arm actuation and combinatorial optimization. We have demonstrated unsupervised clustering and supervised classification in circuit simulations, and have further shown a correspondence between our functional and circuit forms of the AHaH node.

The AHaH node may offer us a building block for a new type of computing with likely application in the field of machine learning. Indeed, we hope that our work demonstrates that functions needed to enable perception (clustering, classification), planning (combinatorial optimization, prediction), control (robotic actuation) and generic computation (universal logic) are possible with a simple circuit that, technologically speaking, may be very close at hand.

## Supporting Information

Video S1 **AHaH clustering demonstration with three Gaussian clusters.**
(MP4)

Video S2 **AHaH clustering demonstration with one Gaussian cluster and one non-Gaussian cluster.**
(MP4)

Video S3 **AHaH clustering demonstration with many Gaussian clusters of various sizes.**
(MP4)

Video S4 **AHaH clustering demonstration with non-stationary clusters.**
(MP4)

Video S5 **AHaH motor control demonstration with 3-joint robotic arm.**
(MP4)

Video S6 **AHaH motor control demonstration with 6-joint robotic arm.**
(MP4)

Video S7 **AHaH motor control demonstration with 9-joint robotic arm.**
(MP4)

Video S8 **AHaH motor control demonstration with 12-joint robotic arm.**
(MP4)

Video S9 **AHaH motor control demonstration with 15-joint robotic arm.**
(MP4)
